# The Sigma‐1 Gene as a Prognostic Marker in Chemotherapy‐Treated Breast Cancer‐Antagonists' Synergism With Paclitaxel In Vitro

**DOI:** 10.1002/cam4.71376

**Published:** 2025-11-12

**Authors:** Preeti Borde, Alia Abdulla, Ebrahim Rajab, Stephen T. Safrany

**Affiliations:** ^1^ Royal College of Surgeons in Ireland Medical University of Bahrain Busaiteen Bahrain; ^2^ School of Medicine, Keele University United Kingdom

**Keywords:** breast cancer, chemotherapy, paclitaxel, sigma‐1 receptor, synergy

## Abstract

**Background:**

Treatment benefits of paclitaxel (Px)‐based chemotherapy are often offset by dose‐limiting side effects. The sigma‐1 receptor (Sig1R) is implicated in chemotherapy (ChT)‐induced neuropathy but its role in the metastatic potential of breast cancer (BCa) has not been properly explored.

**Methods:**

This work investigated the predictive and prognostic value of Sig1R gene (S1R) expression for pathologic complete response (pCR) and distant relapse‐free survival (DRFS) following neoadjuvant ChT (nChT). We further examined the anticancer efficacy of the Sig1R antagonists IPAG and BD1047 in combination with Px in triple‐negative breast cancer (TNBC) cell lines.

**Results:**

We report that S1R positively associated with pCR in two patient cohorts. Upregulated gene clusters in high‐S1R samples of the pCR group are associated with ontology terms related to cell division, DNA replication and microtubule dynamics. High S1R expression was also associated with poor DRFS in TNBC patients. Sig1R knockdown (Sig1R‐KD) in MDA‐MB‐231 and HCC1806 cell lines reduced clonogenic proliferation while treatment with Sig1R antagonists IPAG or BD1047 decreased cell motility. Sig1R‐KD decreased Px‐induced apoptosis; the synergistic effects of Px in combination with IPAG or BD1047 were evaluated by the Chou‐Talalay method. Cytotoxic and antimotility effects of Px were enhanced when combined with Sig1R antagonists.

**Conclusion:**

Taken together, our results indicate that S1R is a potential biomarker for the response to nChT and that targeting Sig1R could enhance Px efficacy while deterring key metastatic mechanisms.

**Abbreviations**
AC: annotation cluster
AUC: area under the curve
BCa: breast cancer
CV: crystal violet
ChT: chemotherapy
CFU: colony‐forming unit
CombI: combination index
DEG: differentially expressed gene
DRFS: distant recurrence‐free survival
DRI: dose‐reduction index
EnR: endoplasmic reticulum
ER: estrogen receptor
Fa: fraction affected
FOVs: fields of view
GO: gene ontology
HER2: human epidermal growth factor receptor 2
HR: hazard ratio
KEGG: Kyoto Encyclopedia of Genes and Genomes
MFE: Mammosphere‐forming efficiency
nChT: neoadjuvant chemotherapy
OR: odds ratio
pCR: pathological complete response
Px: paclitaxel
RD: residual disease
*S1R*
: sigma‐1 receptor (gene)
Sig1R: sigma‐1 receptor (protein)
TNBC: triple‐negative breast cancer
UMAP: uniform manifold approximation and projection
UPR: unfolded protein response

## 1 Introduction

Common chemotherapeutics like paclitaxel (Px) can have dose‐limiting side effects, such as peripheral neuropathy, which severely affect patients' overall quality of life and cause unfavorable effects years after the conclusion of treatment. Dose reduction and/or termination of treatment are common outcomes [1, 2, 3].

The sigma‐1 receptor (Sig1R) is a prolific intracellular chaperone protein expressed in many tissue types and is involved in the regulation of stress response signaling [4, 5]. Early work characterized Sig1R's physiological role as an endoplasmic reticulum (EnR) protein located in microdomains close to the mitochondria. Here, under conditions of cellular stress, Sig1R dissociates from binding immunoglobulin protein (BiP), the master regulator of the cytoprotective unfolded protein response (UPR), and stabilizes type 3 inositol 1,4,5‐trisphosphate receptors thus facilitating Ca^2+^ flow between the EnR and mitochondria [6]. EnR‐related homeostasis is vital for several cellular activities, including proliferation, cell division, and metabolism [7, 8]. Sig1R has been heavily researched in the context of neuropharmacology [9, 10]. The agonist blarcamesine (ANAVEX2‐73) increased plasma amyloid beta (Aβ)42/Aβ40 ratios and slowed the rate of disease progression in Alzheimer's disease patients [11]. The use of Sig1R agonists in many other neurodegenerative disorders has been reviewed elsewhere [9]. Sig1R ligand imaging is furthering our understanding of these disorders [12] as well as being developed to image tumors [13]. In keeping with its cytoprotective role, several studies have shown that a functional Sig1R is a key component of cancer cell machinery and that treatment with Sig1R antagonists reduces cancer cell survival [14, 15, 16]. Cancer cells are strongly dependent on stress response and autophagy to maintain proteostasis; exposure to Sig1R antagonists activates the UPR and autophagic signaling upstream of cell death in several cancer cell lines [17, 18, 19].

Several studies have detailed Sig1R's role in ChT‐associated pain [20, 21, 22]. In a 2015 study by Zhu et al., a rat model of bone cancer pain was used to study the role of increased Sig1R expression in mechanical allodynia. Treatment with the Sig1R antagonist BD1047 significantly alleviated hypersensitivity following mechanical stimulation [23]. Nieto et al. showed that Sig1R‐knockout mice do not develop overt Px‐induced neuropathic pain, nor do they develop cellular irregularities, such as atypical mitochondria, associated with this condition. The genetic ablation of Sig1R mirrored the effects of BD1063 (another Sig1R antagonist) [22, 24]. However, studies on the role of Sig1R antagonists in the actual cytotoxic and anti‐cancer properties of Px have been rare. Cancer mortality is largely driven by metastatic spread, in which cells of the primary tumor migrate from the primary site and disseminate to distant sites. We have previously shown that both Sig1R gene (*S1R*) and protein (Sig1R) expression positively correlate with poor patient survival and that TNBC samples express high levels of *S1R* compared to other breast cancer (BCa) subtypes [19]. The present study was undertaken to investigate whether *S1R* expression can be used to distinguish prognosis‐relevant subgroups within ChT‐treated patients. We further examined whether combination with the Sig1R antagonists BD1047 and IPAG enhances the cytotoxic effects of Px in TNBC cell lines.

## 2 Material and Methods

### 2.1 GEO Patient Datasets

Clinical characteristics and tissue gene expression data for BCa patients who received neoadjuvant ChT (nChT) and had documented pCR/RD as well as DRFS data (GSE25066; *n* = 508, the training cohort) [25] were downloaded from the Gene Expression Omnibus (GEO) using the GEOquery R package. Data for the validation cohort GSE20194 (*n* = 278) [26], was similarly obtained. *Z*‐scores were calculated using means and standard deviations in the training set. UMAP plots and Venn diagrams were generated using the GEO2R web tool (https://www.ncbi.nlm.nih.gov/geo/geo2r/). Differentially expressed genes (DEGs) were identified in GEO2R using the Benjamini & Hochberg (false discovery rate) method for multiple testing (adjusted *p*‐value, *p*_adj_ < 0.05). DEGs were filtered on their log2‐fold change (log_2_FC) values. A log_2_FC > 0.6 was considered upregulated, and a log_2_FC < −0.6 was considered downregulated. Enrichment analyses, including GO and KEGG analyses, were performed using the DAVID v6.7 web tool [27].

### 2.2 Cell Culture

Cell lines were purchased from Angioproteomie (Boston, MA, USA). MDA‐MB‐231 and HCC1806 cells were respectively cultured in DMEM (D5796, Merck, Germany) and RPMI (A10491, Gibco, UK) media supplemented with 10% FCS. Cells were maintained in a humidified incubator at 37°C and 5% CO_2_, were not cultured for more than 20 consecutive passages and were regularly tested for mycoplasma contamination.

### 2.3 Drug Treatments

Px, N‐[2‐(3,4‐dichlorophenyl)ethyl]‐N‐methyl‐2‐(dimethylamino)ethylamine dihydrobromide (BD1047), and 1‐(4‐iodophenyl)‐3‐(2‐adamantyl)guanidine (IPAG) were purchased from Tocris Bioscience (Bristol, UK) and stocks were made in DMSO. Cells were serum‐starved overnight prior to treatment, and final drug concentrations were obtained by diluting stock solutions in 5% FCS supplemented basal media.

### 2.4 Drug Synergy Assays

MDA‐MB‐231 or HCC1806 cells were seeded in 96‐well plates (2500 cells/well) and treated with Px (0, 0.0001, 0.001, 0.01, 0.1, 1.0 μM) in combination with IPAG (0, 1.0, 2.0, 5.0, 10.0 μM) or BD1047 (0, 10.0, 20.0, 50.0, 100.0 μM) for 72 h. Cell viability was measured by MTS assays (Promega, USA), and possible synergy between drug pairs was analyzed by the Chou–Talalay method [28]. The combination index (CombI), fraction affected (Fa) and dose‐reduction index (DRI) were calculated with CompuSyn software (ComboSyn Inc., Paramus, USA).

### 2.5 Sig1R Knockdown

MDA‐MB‐231 and HCC1806 cells were seeded in 6‐well plates (150,000 cells/well) and transfected with 10 nM of either *S1R* or non‐targeted SMARTpool siRNA (ON‐TARGETplus, Dharmacon, USA) using JetPrime transfection agent (PolyPlus, Germany). Forty‐eight hours later, cells were seeded into plates for the experiments detailed below and re‐transfected (except for mammospheres, which were plated after a single transfection). Sig1R knockdown (Sig1R‐KD) was confirmed by western blotting. Briefly, protein lysates from transfected cells were subjected to SDS‐PAGE and transferred to nitrocellulose membranes (GE Healthcare, UK), which were subsequently blocked and incubated with primary antibodies in 5% non‐fat milk. The antibodies used were Sig1R (1.0 μg/mL, Santa Cruz Biotechnology, #SC137075) and β‐actin (0.2 μg/mL, Merck, #A2228).

### 2.6 Propidium Iodide Staining

Transfected cells were seeded in 96‐well plates (3000 cells/well for MDA‐MB‐231 cells and 5000 cells/well for HCC1806 cells). Following 24‐h drug treatments, the cells were washed with PBS and incubated with 5 μL/mL Hoechst 33342 nuclear stain (BD Biosciences, Germany) for 1 h at 37°C and 5% CO_2_. Cells were labeled with 5 μL/mL propidium iodide (PI) (Abcam, USA) for 10 min and imaged at 10× using a fluorescent inverted microscope. At least three fields of view (FOVs) were captured per treatment condition, and images were processed using ImageJ (NIH, Bethesda, MD, USA). Nuclei (labeled with Hoechst, blue channel) and nuclei positive for PI (red channel) were counted, and the apoptotic fraction expressed as the number of nuclei positive for PI/total number of nuclei (PI^+^/nuclei).

### 2.7 Mammosphere Assay

Mammospheres were cultured in complete MammoCult medium (Stem Cell Technologies, Vancouver, BC, Canada) according to the manufacturer's instructions. Briefly, transfected MDA‐MB‐231 and HCC1806 cells were gently syringed to make clump‐free, single‐cell suspensions. Cells were seeded (1000 cells/well) in ultra‐low attachment 6‐well plates (Corning). Mammospheres were allowed to grow undisturbed for 5–6 days and then counted (≥ 50 μm) and photographed. Mammosphere‐forming efficiency (%MFE) of each condition was calculated as [number of mammospheres counted per well/number of cells seeded per well] × 100.

### 2.8 Colony‐Forming Unit Assay

Transfected MDA‐MB‐231 and HCC1806 cells were plated (1000 cells/well) in 6‐well plates and allowed to attach. The media was changed 24 h later, and the colonies were allowed to form for 10 days with media being refreshed every 3–4 days. The cells were then washed gently with PBS and stained with 0.4% crystal violet (CV) for 1 h at RT. CV‐stained plates were dried overnight, the dye from each well was dissolved in methanol, and the absorbance of the CV solution from each described condition was measured at 592 nm in triplicate using a spectrometer.

### 2.9 Scratch Assay

Uniform scratches were made in a monolayer of confluent MDA‐MB‐231 cells seeded in 12‐well plates and imaged at 0 h. Cells were incubated with treatment media and serial photographs of the scratch at three different FOVs per well captured for up to 48 h. Linear width between the edges of the scratch at least 3 random positions per FOV was measured in ImageJ for each timepoint. The relative %reduction in the scratch width was calculated as [(width_T0_—width_Tx_)/(width_T0_)] × 100.

### 2.10 Statistical Analyses

Statistical analyses were performed using GraphPad Prism (GraphPad Software, San Diego, CA, USA) unless otherwise stated. In vitro data are representative of at least three independent biological replicates and are reported as replicate means ± SEM unless otherwise stated. Specific tests applied are detailed in each figure legend. *p*‐values < 0.05 were considered significant.

## 3 Results

### 3.1 Sigma‐1 Receptor Gene *(S1R)* Expression as a Predictive Factor for pCR

**FIGURE 1:**
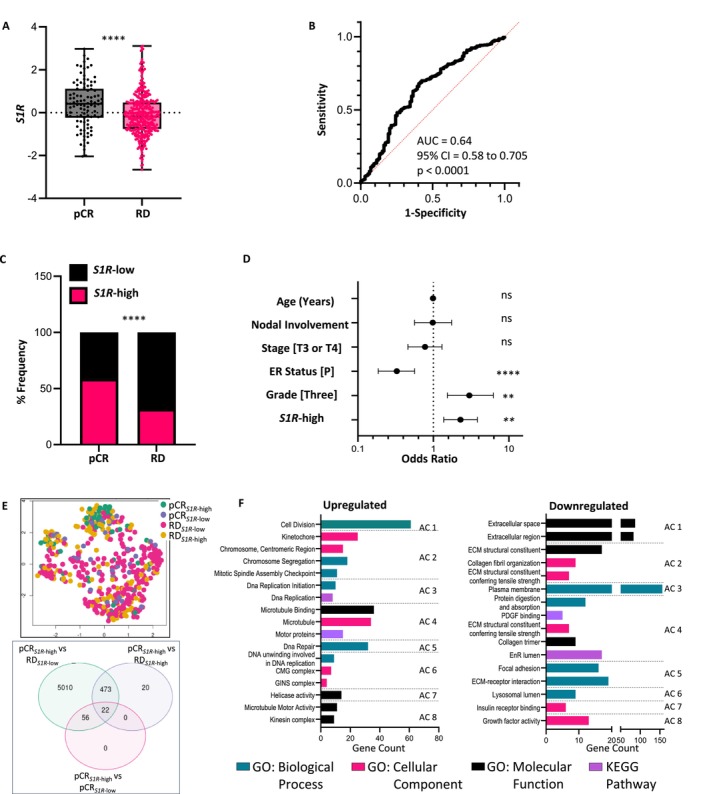
*S1R* as a predictor of response following nChT. (A) Box and whisker plot depicting the difference in *S1R* expression between the pCR and RD groups in the training (GSE25066, *n* = 508) cohort. Statistical analysis was performed with Welch's *t*‐test. (B) ROC curve depicting the utility of *S1R* expression for discriminating between pCR and RD patients. (C) %Frequency distribution of dichotomized *S1R*‐low (*S1R*‐low) and high (*S1R*‐high) expressing samples between the pCR and RD groups in the training cohort. The statistical analysis was performed with Fisher's exact test. (D) Forest plot displaying the multiple logistic regression ORs (±95% CI) for pCR according to clinicopathological variables and *S1R*‐high status in the training cohort. (E) GEO2R‐generated UMAP plot of the patterns of separability between samples classified into 4 groups: pCR_*S1R*‐high_, RD_*S1R*‐high_, pCR_*S1R*‐low_, and RD_*S1R*‐low_. GEO2R‐generated Venn diagram of common and unique DEGs in the pCR_*S1R*‐high_ group versus other groups. (F) GO functional (3 categories) and KEGG enrichment analyses from DAVID of up‐ and downregulated genes from the pCR_*S1R*‐high_ versus RD_*S1R*‐low_ group. Graphs show the pathways in the top 8 significant annotation clusters with the number of genes involved in the respective pathway. AC, annotation cluster; ECM, extracellular matrix; EnR, endoplasmic reticulum; PDGF, platelet‐derived growth factor; OR, odds ratio. ns: *p* > 0.05, **p < 0.01, ****p < 0.0001.

As we had previously observed [19], *S1R* expression was greater in the tumors of patients who achieved pCR than in those of patients who did not (*p* < 0.0001) (Figure 1A). In the training cohort (GSE25066), the ability of *S1R* expression to distinguish between these two groups was evaluated by receiver operating characteristic (ROC) analysis, which revealed an area under the curve (AUC) of 0.64 (95% confidence intervals (95% CI), 0.58 to 0.705; *p* < 0.0001). An optimal cutoff with a sensitivity of 68% (95% CI 0.63 to 0.73) and a specificity of 60% (95% CI 0.50 to 0.69) was obtained by calculating the maximum Youden index. With this cutoff, samples were dichotomized into *S1R*‐low (*n* = 321) and *S1R*‐high (*n* = 187). The majority of pCR samples were *S1R*‐high (58.6%) and most RD samples *S1R*‐low (68.1%) (*p* < 0.0001) (Figure 1B,C). We next performed multiple logistic analyses using various clinicopathological factors and dichotomized *S1R*‐low/high status to predict pCR in the training cohort. We found the *S1R*‐high classification to be a favorable factor (odds ratio (OR) ≥ 2.0) associated with pCR outcome (OR = 2.28, 95% CI 1.37 to 3.82; *p* = 0.0015) along with high grade (OR = 2.99, 95% CI 1.53 to 6.22) and estrogen receptor‐negative (ER_neg_) status (OR = 3.06, 95% CI 5.38 to 1.83) (Figure 1D).

The uniform manifold approximation and projection (UMAP) generated by the GEO2R online tool demonstrated that the pCR_*S1R*‐high_, RD_*S1R*‐high_, pCR_*S1R*‐low_, RD_*S1R*‐low_ grouping had appreciable discriminability, as most pCR_*S1R*‐high_ samples were assigned to the same cluster (Figure 1E). To identify differentially expressed genes (DEGs) in the pCR_*S1R*‐high_ set which we assigned as the “good‐outcome” phenotype tumors, we used GEO2R to intersect DEGs from this group on a Venn diagram with those from the other three groups. The pCR_*S1R*‐high_ vs. RD_*S1R*‐low_ gene signature contained 5010 unique DEGs (Figure 1E). Gene Ontology (GO) and Kyoto Encyclopedia of Genes and Genomes (KEGG) analyses of the upregulated (*n* = 846) and downregulated (*n* = 548) genes were performed with the Database for Annotation, Visualization and Integrated Discovery (DAVID) (Table S1). The significant (adjusted *p* value, *p*_adj_ < 0.05) terms in the top 8 (ranked by enrichment scores) annotation clusters (ACs) are shown in Figure 1F. GO analysis suggested that most upregulated DEGs in the biologically relevant clusters were enriched in pathways involved in cell division and microtubule dynamics. KEGG annotation analysis revealed that the upregulated DEGs were significantly enriched in DNA replication. The downregulated genes were mostly associated with the following GO terms: extracellular space, extracellular region, plasma membrane, and ECM receptor interaction. KEGG annotation of the downregulated genes revealed platelet‐derived growth factor binding and the EnR lumen (Figure 1F). There were not enough unique genes in the pCR_*S1R*‐high_ versus RD_*S1R*‐high_ group (*n* = 20) to perform meaningful DEG analysis.

We tested for associations between *S1R* status and pCR in a separate validation cohort (GSE20194) for whom pCR and RD statuses were available. Because the training cohort consisted largely of human epidermal growth factor receptor 2‐negative (Her2_neg_) samples (~95.5%), we focused on the Her2_neg_ group in the validation cohort (*n* = 219). We found that *S1R*‐high status, as per the cutoff established in the training cohort, was again associated with more frequent pCR than was RD (44.1% vs. 10.8%, *p* < 0.0001), and that *S1R*‐high classification was also an independent factor in a model predicting pCR (OR = 2.88, 95% CI 1.09 to 7.58; *p* = 0.03), as was high grade (OR = 4.07, 95% CI 1.30 to 15.50) and ER_neg_ status (OR = 4.67, 95% CI 1.745 to 13.77) (Figure S1A,B).

We did not observe similar associations in the ER_pos_Her2_neg_ or TNBC patients in either cohort (data not shown).

### 3.2 *S1R* Expression as a Prognostic Factor for Distant Relapse‐Free Survival Following Residual Disease in TNBC

**FIGURE 2:**
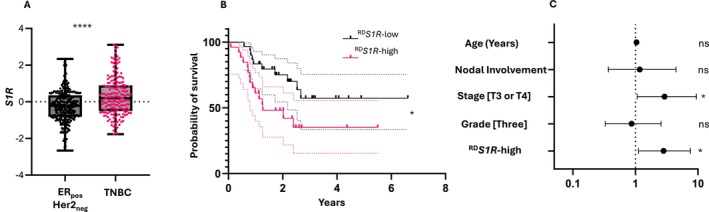
*S1R* as a prognostic factor for distant relapse‐free survival in TNBC patients with residual disease following nChT. (A) Box and whisker plot depicting the distribution of *S1R* expression between ER_pos_Her2_neg_ and TNBC tumor samples. Statistical analysis was performed with Welch's *t*‐test. (B) Kaplan–Meier curves for associations between *S1R* status and DRFS in TNBC patients with post‐nChT RD (^RD^ *S1R*‐high/low) in the training cohort. Statistical analysis was performed with the log‐rank *t*‐test. (C) Forest plots displaying the HR (±95% CI) for DRFS in a multivariable Cox regression model for TNBC patients with post‐nChT RD according to clinicopathological variables and ^RD^ *S1R*‐high status. ns: *p* > 0.05, *p < 0.05, ****p < 0.0001.

As we have observed in other datasets [19], samples in the TNBC cohort had significantly greater *S1R* expression (Figure 2A) but ROC analysis could not distinguish between patients who experienced distant relapse or death in this group (AUC = 0.54, 95% CI 0.45 to 0.64; *p* = 0.4). Nevertheless, incorporating *S1R* expression as a continuous variable in multivariate analysis, showed that it was associated with DRFS in TNBC patients within the RD subgroup (Hazard ratio (HR) = 1.40, 95% CI 1.06 to 1.83; *p* = 0.02) (data not shown) indicating there is a threshold value for predicting DRFS outcome. Hence, we divided TNBC patient samples into quartiles by *S1R* expression. The samples from the highest expression quartile (^RD^ *S1R*‐high) subgroup had worse DRFS than the lowest (^RD^ *S1R*‐low) (HR = 2.53, 95% CI 1.12 to 5.65; *p* = 0.015) (Figure 2B). This association remained significant in multivariate Cox analysis adjusting for age, nodal involvement, stage, and grade as confounding factors (HR = 2.82, 95% CI 1.12 to 7.58; *p* = 0.03) (Figure 2C). No similar associations were detected in the ER_pos_Her2_neg_ group (data not shown). Determining prognostic factors in the pCR group was difficult due to the expected small number of distant recurrence events.

### 3.3 Sig1R Depletion or Pharmacological Inhibition Reduces the Metastatic Potential of TNBC Cell Lines

**FIGURE 3:**
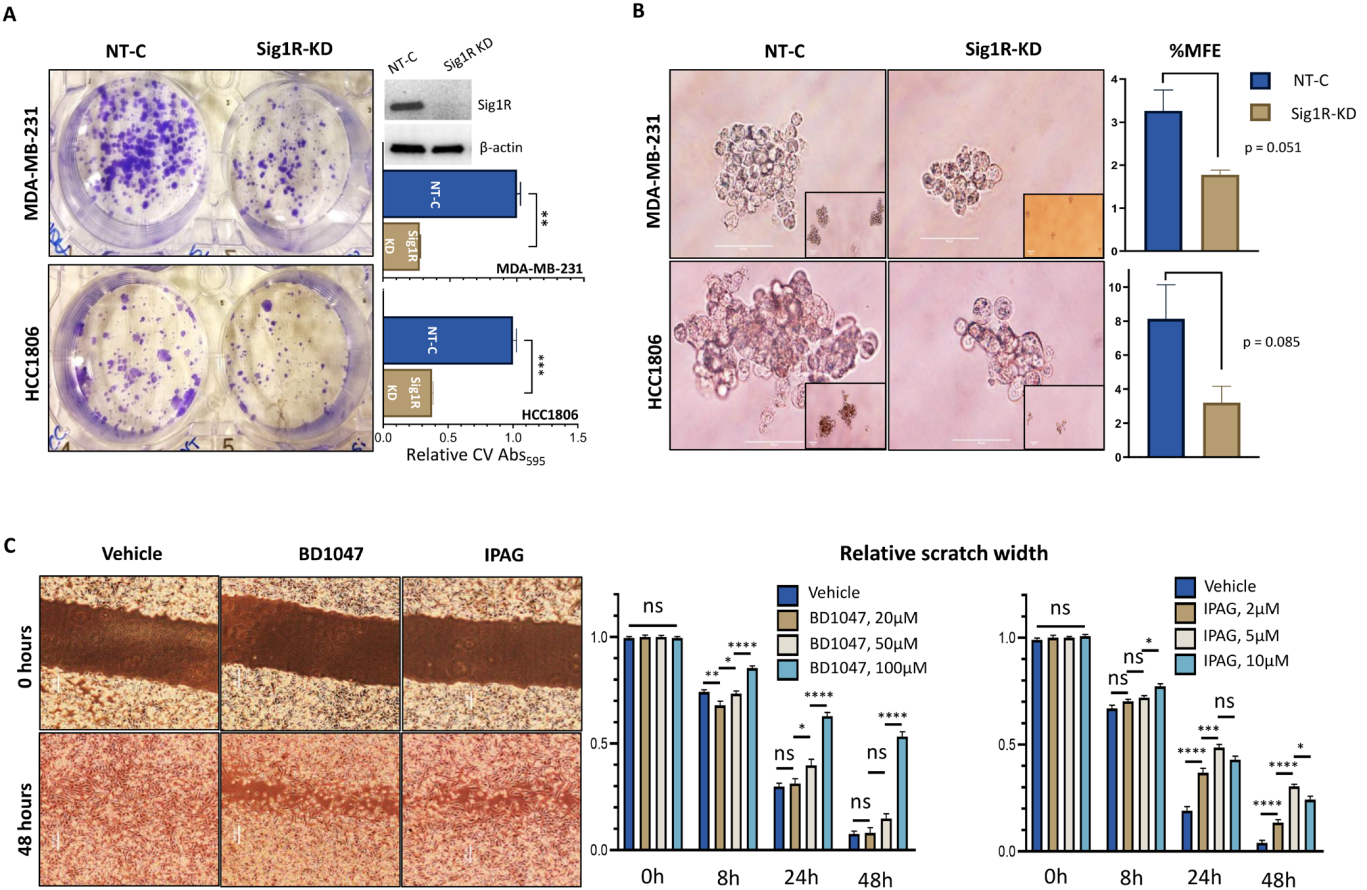
Sig1R is needed for anchorage‐independent growth, clonogenic proliferation and motility in TNBC cell lines. (A) Representative images showing the effects of Sig1R‐KD on the CFU potential of the MDA‐MB‐231 (*n* = 5) and HCC1806 (*n* = 3) cell lines. A western blot for Sig1R‐KD is shown for MDA‐MB‐231 cells; the full, uncropped, image can be found in Figure S2. The graphs represent the average CV absorbance relative to that of NT‐C. Statistical analysis was performed with Welch's *t*‐test. (B) Representative images showing the effects of Sig1R‐KD on the %MFE of MDA‐MB‐231 (*n* = 4) and HCC1806 (*n* = 4) cells. Images were taken at 40×. The insert images are shown at 10×; the scale bars denote 50 μm. The graphs compare the %MFE of NT‐C and Sig1‐KD cells. Statistical analysis was performed with Welch's *t*‐test. (C) Representative images showing the migration of cells into the scratch when treated with increasing concentrations of IPAG (*n* = 3) or BD1047 (*n* = 3) at 8, 24 and 48 h. The graphs present the relative (to time = 0 h, 0 h) mean scratch width at each timepoint. Statistical analysis was performed by one‐way ANOVA with Tukey's post hoc test. ns: *p* > 0.05, *p < 0.05, **p < 0.01, ***p < 0.001, ****p < 0.0001.

We investigated whether Sig1R plays a role in anchorage‐independent growth, clonogenic proliferation and motility. CV staining of both MDA‐MB‐231 (*p* = 0.001) and HCC1806 (*p* = 0.0009) cells revealed that colony‐forming unit (CFU) capability was significantly lower in Sig1R‐knockdown (Sig1R‐KD) cells than in nontargeted siRNA controls (NT‐C) (Figure 3A). Furthermore, Sig1R‐KD also reduced the %MFE of MDA‐MB‐231 (3.3% vs. 1.8%, *p* = 0.051) and HCC1806 cells (8.1% vs. 3.2%, *p* = 0.085), although these reductions did not meet the threshold of statistical significance in either cell line (Figure 3B). Wound healing assays were performed to evaluate the motility of MDA‐MB‐231 cells following exposure to the Sig1R antagonists IPAG and BD1047. We found a reduction in cell migration into the gap over 48 h for both BD1047‐ and IPAG‐treated cells (Figure 3C).

### 3.4 Synergism Between Px and Sig1R Antagonists

**FIGURE 4:**
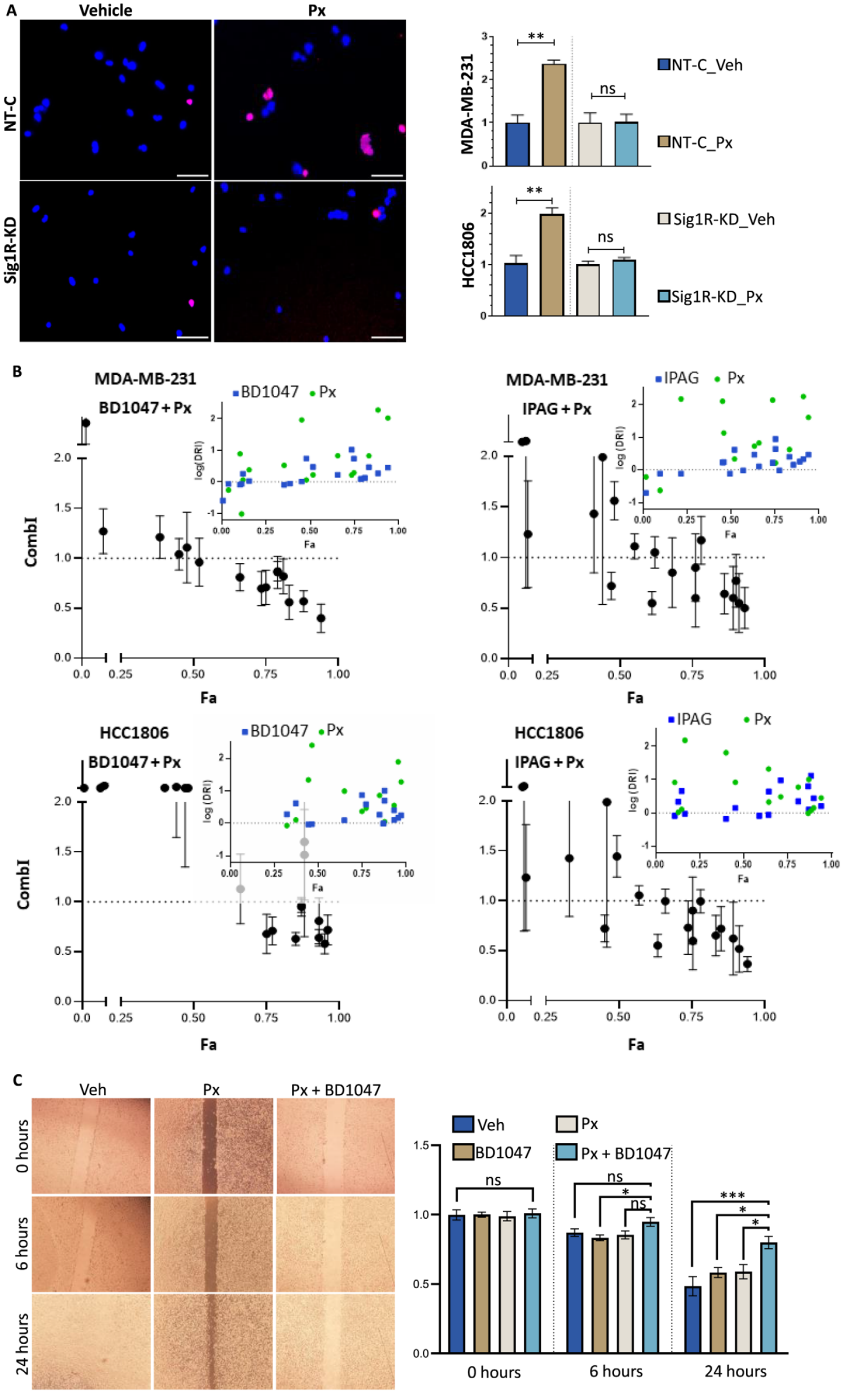
Synergism between Px and Sig1R antagonists. (A) Representative images of Hoechst 33342 (blue channel) and PI (red channel) double staining of NT‐C or Sig1R‐KD MDA‐MB‐231 (*n* = 5) and HCC1806 (*n* = 4) cells treated with Px for 24 h. Images were captured at 10×, and the scale bars denote 50 μm. The graphs represent the fraction of PI‐positive nuclei (PI^+^/nuclei) counted in each treatment condition for Px. The readings were normalized to those of vehicle (Veh)‐treated cells. Statistical analysis was performed with Welch's *t*‐test. (B) MDA‐MB‐231 and HCC1806 cells were treated simultaneously with five concentrations of Px and four concentrations of IPAG or BD1047 alone or in combination with each of the other two drugs (*n* = 4). Drug interactions were assessed by the Chou‐Talalay method. Fa‐CombI plots of the effects of drug combinations on cell viability. A CombI < 1 indicates synergism, a CombI = 1 indicates additivity, and a CombI > 1 indicates antagonism. The inserts depict Fa‐DRI plots; log(DRI) = 0 indicates no dose reduction; log(DRI) > 0 indicates favorable dose reduction; log(DRI) < 0 indicates unfavorable dose reduction. (C) Representative images showing the migration of MDA‐MB‐231 cells into a scratch after 24 h of treatment with Px (0.1 μM) or BD1047 (100.0 μM) alone or in combination (*n* = 4). The graphs present the relative (to 0 h) mean scratch width at each timepoint. Statistical analysis was performed by one‐way ANOVA with Tukey's post hoc test. ns: *p* > 0.05, *p < 0.05, **p < 0.01, ***p < 0.001.

To investigate whether ChT‐induced apoptosis is mediated by Sig1R, we treated Sig1R‐KD MDA‐MB‐231 and HCC1806 cells with 0.1 μM Px for 24 h. As measured by the fraction of PI^+^ nuclei relative to the total number of nuclei (PI^+^/nuclei), Sig1R‐KD reduced apoptosis in Px‐treated cells. In MDA‐MB‐231 cells, the proportion of apoptotic cells was 2.4 times greater in the NT‐C Px‐treated cells than in the vehicle‐treated controls (*p* = 0.002). There was no increase in the apoptotic fraction in Px‐treated Sig1R‐KD cells (*p* = 0.94). The results in HCC1806 cells were similar to those observed with MDA‐MB‐231 cells (Figure 4A). The cytotoxic effect of Px in combination with IPAG and BD1047 was measured by MTS viability assays. MDA‐MB‐231 and HCC1806 cells were treated with five concentrations of Px and four concentrations of IPAG or BD1047 in every possible combination. CompuSyn software was used to calculate the combination index (CombI) of the drugs by the Chou–Talalay method [28]. Fraction affected (Fa)–CombI plots of the effects of drug combinations on cell viability are shown in Figure 4B. CombI < 1, CombI = 1, and CombI > 1 represent synergistic, additive, and antagonistic effects, respectively. The dose reduction index (DRI) indicates the fold reduction in the dose of one drug in a synergistic combination that can produce the same effect as treatment with each drug alone. For Fa levels higher than 0.5, the CombI for combinations of IPAG/BD1047 + Px in both cell lines is shown in Table S2. Several combinations showed obvious synergism (CombI < 0.7 [29]) such as 10.0 μM IPAG + 10.0 nM Px in HCC1806 (Fa > 0.9, CombI = 0.52 ± 0.15) or 100.0 μM BD1047 + 1.0 nM Px in MDA‐MB‐231 (Fa > 0.8, CombI = 0.56 ± 0.12) (Table S2). We also investigated the functional effects of Px (0.1 μM) with and without combination with BD1047 (100.0 μM) on motility by scratch assays in MDA‐MB‐231 cells. The average scratch width reduction was 22% for BD1047 + Px‐treated cells, 40% for Px‐treated cells (*p* = 0.04) and 42% for BD1047‐treated cells (*p* = 0.03) (Figure 4C).

## 4 Discussion

Few previous studies have performed gene enrichment analysis of *S1R* in BCa. We determined that most upregulated genes in the ‘good outcome’ tumors were enriched in pathways involved in cell division, microtubule dynamics, and DNA repair. In broad agreement with our results, examination of a small cohort of colon adenocarcinoma samples revealed that *S1R* was part of a module of genes enriched in cell division‐ and proliferation‐related terms including RNA polymerase, nucleotide metabolism (in KEGG) and translation, and ribosome (in GO) [30]. A prostate cancer study showed that *S1R* was enriched in tumors with high adipogenesis and ROS‐related gene sets [31].

Gueguinou et al. showed that the Sig1R ligand igmesine decreased motility in the colorectal cell line HCT116 and in MDA‐MB‐435S, a cell line originally thought to be of BCa but since shown to be of melanocytic origin [32]. Gene silencing of Sig1R also attenuated motility in colorectal cancer cells [33]. We have shown that Sig1R‐KD also stunts other key measures of BCa metastatic potential—clonogenic proliferation and anchorage‐independent growth capacity. One previous study reported that haloperidol, a non‐specific Sig1R antagonist, had an additive effect at a single concentration tested, with ChT drugs including Px in a BCa cell line; synergy calculations were not reported [34]. IPAG is an extensively studied Sig1R antagonist, and modified versions with better in vivo physicochemical properties are well tolerated in animal models [16]. BD1047 by itself is not a potent inducer of apoptosis [35] but given its desirable profile in alleviating pain [36, 37, 38], its anticancer effects in combination with other drugs deserve intense investigation. Our results show that IPAG/BD1047 combinations with Px show synergistic cytotoxicity at several concentrations.

It is not clear what mechanistic overlaps exist between Sig1R signaling and Px cytotoxicity. Px is well known to act by stabilizing microtubules, thus causing cell cycle arrest, mitotic catastrophe, and ultimately cell death [39]. Tsai et al. demonstrated that knocking out neuronal Sig1R increases tau hyperphosphorylation and results in the production of sparser, shorter axons, suggesting its role in regulating neuronal microtubule dynamics [40]. A tumor cell's capacity to resist apoptosis and proliferate by progressing through the cell cycle is strongly dependent on cell volume regulation through transmembrane voltage‐gated ion channels [41, 42]. In small cell lung cancer and leukemia cells, Sig1R agonists cause the accumulation of p27^Kip1^ and a decrease in cyclin A (negative and positive influencers of G1/S transition respectively) [43], partly through functional inhibition of voltage‐regulated K^+^ channels [44]. Similarly, Sig1R agonists inhibited volume‐regulated Cl^−^ channels, and Sig1R overexpression conferred resistance to apoptotic volume decrease [45]. At least one study has shown that Sig1R antagonists cause shrinkage of cancer cells [17] and Sig1R has been proposed to promote apoptosis resistance in cancer by exerting a tonic clamp of volume‐regulated K^+^ and Cl^−^ channels, which prevents apoptotic volume decrease without affecting regulatory volume decrease; the latter being required for cell division [45, 46].

Fluvoxamine, a widely used antidepressant and a Sig1R agonist, stimulated Sig1R expression in neuroblastoma cells and alleviated Px‐induced, EnR stress‐mediated cell death; treatment with a Sig1R antagonist reversed these protective effects [47, 48]. Fluvoxamine‐induced cytoprotective Sig1R overexpression is mediated by increased activating transcription factor‐4 (ATF4), a transcription factor activated in cellular stress, interestingly without triggering larger UPR signaling. Treatment with a Sig1R antagonist blocked fluvoxamine‐induced ATF4 upregulation [47].

While our short‐term (24 h) treatment in Sig1R‐KD cells showed that Px‐cytotoxicity is mediated in part through Sig1R, ligand‐bound Sig1R exists in various oligomeric forms [49, 50] and its functional protein–protein interactions in cancer are likely to be complex. ATF4 is necessary for Px‐induced autophagy and the development of apoptosis‐resistance [51]. It is possible that Sig1R antagonists enhance the anticancer properties of Px through combined cell division dysregulation and downregulation of ATF4. However, the stress‐induced translation of ATF4 is common to the UPR as well as to noncanonical EnR stress programs such as the integrated stress response (ISR) [52] and preclinical clarification of Sig1R's role in the latter is necessary. While Sig1R is known to interact with UPR proteins such as BiP and IRE1 [53, 54], its role in the PERK‐ATF4 pathway is relatively undescribed. Further, given its well‐documented role in the UPR, prolonged Sig1R antagonism may simply potentiate Px cytotoxicity through sustained UPR activation, which ultimately turns cytotoxic [55].

One of the limitations of our current work is that it has only performed gene enrichment/depletion analysis on samples from a single dataset (GSE25066). The modest AUC we report (Figure 1) does not meet the threshold for clinical utility; nonetheless we do show that binarizing patients based on this cutoff demonstrated high‐*S1R* status to be an independent predictor for nChT success in two cohorts. Px is also heavily utilized in treating Her2_pos_ BCa. Due to the small number of patients with Her2_pos_ tumors (*n* = 6 in GSE25066 and *n* = 59 in GSE20194), we could not carry out in‐depth investigation in this subtype. Sig1R regulates several proteins key to tumor aggressiveness and treatment resistance, including immune checkpoint inhibitors and UPR initiators [53, 56]. Uncovering a ‘high‐*S1R* gene signature’ may reveal subtype‐specific identification of post‐nChT good outcome groups. Further, while Sig1R antagonists are being studied in the context of taxane and platinum‐based ChT, its role in doxorubicin‐induced neuropathy is largely unexplored [20, 21, 22, 57]. The patients in the two cohorts used in this study received time and dose treatment regimens of different taxanes (Px or docetaxel) with anthracycline combinations. Thus, evaluating ChT outcomes and DEGs from the intersections of several appropriate datasets could reveal comprehensive hubs of *S1R* involvement while controlling for tumor heterogeneities and co‐treatments.

To the best of our knowledge, the current study is the first analysis of *S1R* as a biomarker for the nChT response in BCa and the first in vitro investigation to show synergy between Sig1R antagonists and Px in BCa; hence our results provide a strong preclinical basis to investigate in vivo therapeutic value. While pharmacokinetics of cellular accumulation and cytotoxicity are bound to be different from in vitro, other work has successfully demonstrated in vivo efficacy with similar parameters as ours [58, 59, 60]. For instance, Xu et al. showed in vitro synergism (CombI < 0.7) between a Bcl‐2 inhibitor and the ChT drug irinotecan and that this combination reduced tumor size in a mouse model of non‐small cell lung cancer [60]. An in vivo animal model of BCa metastasis would confirm the synergism between Sig1R antagonists and Px while providing opportunities to study the mechanistic stress response pathways involved upstream and downstream of cytotoxicity. Because the UPR and ISR are both vital to the physiological stress response, the results of the few previous studies on Sig1R agonist‐mediated alleviation of stress‐induced neurotoxicity without involvement of the UPR require resolution [47]. This could be done through Sig1R‐knockout models and small molecule inhibitors/activators specific to the ISR [61, 62]. However, given Sig1R's role in neurologic disorders and ChT‐induced pain [22], any in vivo study on synergistic Sig1R antagonists + Px treatment should incorporate behavioral models for Px‐induced neuropathy (such as mechanical allodynia or thermal hyperalgesia) to provide measures of pain sensitivity and other side effects. Such measures were beyond the facilities of the current study but offer compelling directions for future work. We expect that combining Px and Sig1R antagonists would reduce Px‐associated off‐target effects by decreasing the required dosage while preserving clinical efficacy.

## Author Contributions

**Preeti Borde:** conceptualization, investigation, formal analysis, visualization, writing – original draft. **Alia Abdulla:** investigation, writing – review and editing. **Ebrahim Rajab:** funding acquisition, supervision, writing – review and editing. **Stephen T. Safrany:** conceptualization, supervision, visualization, writing – review and editing.

## Ethics Statement

The authors have nothing to report.

## Consent

The authors have nothing to report.

## Conflicts of Interest

The authors declare no conflicts of interest.

## Supporting information


**Figure S1:** (A) %Frequency distribution of dichotomized samples between the pCR and RD groups in samples from the validation (GSE20194) cohort. Statistical analysis was performed with Fisher's exact test. (B) Forest plot displaying the multiple logistic regression ORs (±95% CI) for pCR according to clinicopathological variables and *S1R*‐high status. ns: *p* > 0.05, *p < 0.05, **p < 0.01, ****p < 0.0001.

**Figure S2:** Western blot evaluation of sigma‐1 receptor knockdown (Sig1R‐KD) following transfection with *S1R* siRNA or non‐targeted siRNA controls (NT‐C).

**Table S1:** Genes upregulated (log_2_FC > 0.6) and downregulated (log_2_FC < −0.6) in the pCR_*S1R*‐high_ versus RD_*S1R*‐low_ group (GSE25066).

**Table S2:** Calculated values for combination indices (CombI) and fraction affected (Fa) as described by the Chou‐Talalay method on MDA‐MB‐231 and HCC1806 cells exposed to Sigma‐1 receptor antagonists IPAG/BD1047 plus Px.

## Data Availability

The data that support the findings of this study are available from the corresponding author upon reasonable request.
