# Supplementary material for: The Sigma‐1 Gene as a Prognostic Marker in Chemotherapy‐Treated Breast Cancer‐Antagonists' Synergism With Paclitaxel In Vitro
Source: Cancer Med. 2025 Nov 12;14(21):e71376. doi: 10.1002/cam4.71376 (PMC12611308; doi:10.1002/cam4.71376)

Supplementary Figure 1:

(A) %Frequency distribution of dichotomized samples between the pCR and RD groups in samples from the validation (GSE20194) cohort. Statistical analysis was performed with Fisher's exact test. **(B)** Forest plot displaying the multiple logistic regression ORs (±95% CI) for pCR according to clinicopathological variables and *S1R*-high status in the validation cohort.

A

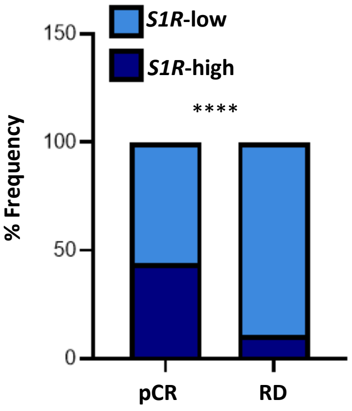

B

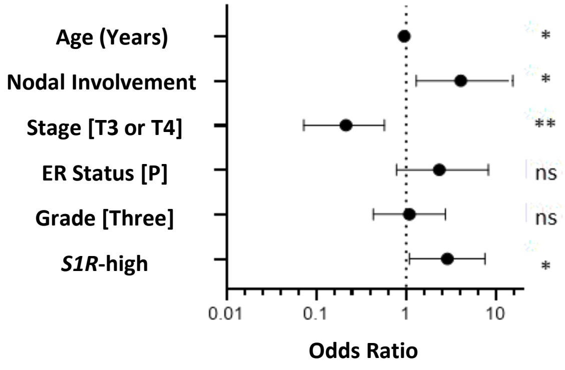

Supplement: Supplementary file 1 — Figure S1: (A) %Frequency distribution of dichotomized samples between the pCR and RD groups in samples from the validation (GSE20194) cohort. Statistical analysis was performed with Fisher's exact test. (B) Forest plot displaying the multiple logistic regression ORs (±95% CI) for pCR according to clinicopathological variables and S1R‐high status. ns: p > 0.05, *p < 0.05, **p < 0.01, ****p < 0.0001. [file CAM4-14-e71376-s001.pdf]
