# Supplementary material for: The Sigma‐1 Gene as a Prognostic Marker in Chemotherapy‐Treated Breast Cancer‐Antagonists' Synergism With Paclitaxel In Vitro
Source: Cancer Med. 2025 Nov 12;14(21):e71376. doi: 10.1002/cam4.71376 (PMC12611308; doi:10.1002/cam4.71376)
Supplement: Supplementary file 2 — Figure S2: Western blot evaluation of sigma‐1 receptor knockdown (Sig1R‐KD) following transfection with S1R siRNA or non‐targeted siRNA controls (NT‐C). [file CAM4-14-e71376-s002.pdf]

Supplementary Figure 2:

Western blot evaluation of sigma-1 receptor knockdown (Sig1R-KD) following transfection with *S1R* siRNA or non-targeted siRNA controls (NT-C).

Sig1R

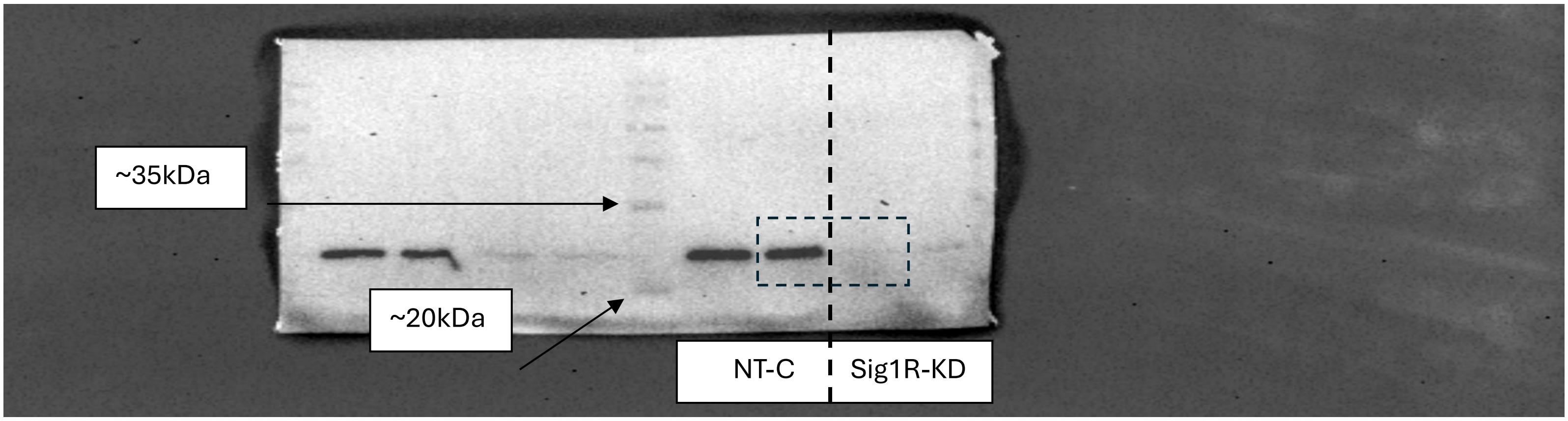

β-actin

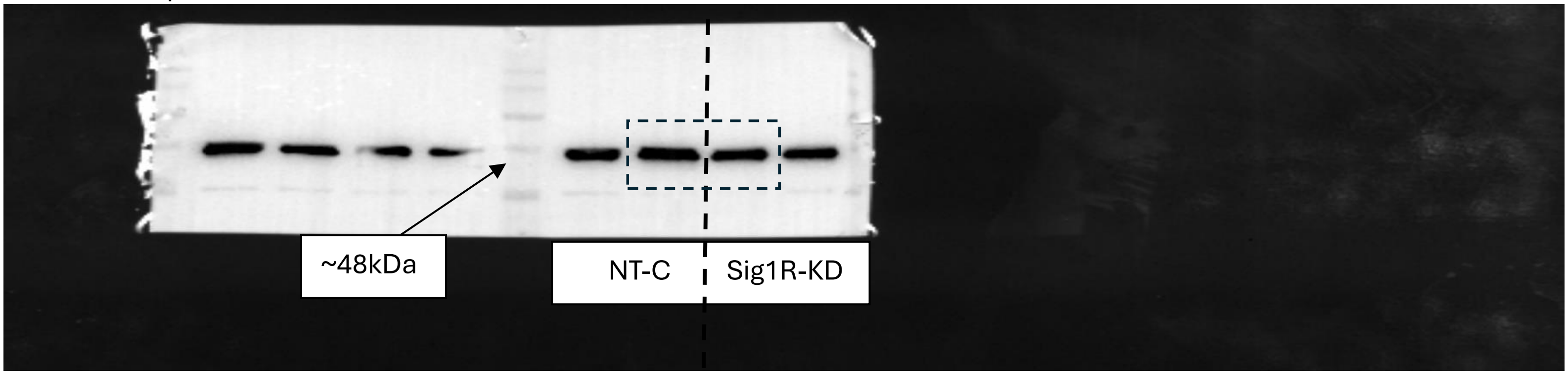

The cropped sections used in Figure 3 are highlighted in the dotted rectangles.
