# Supplementary material for: The Sigma‐1 Gene as a Prognostic Marker in Chemotherapy‐Treated Breast Cancer‐Antagonists' Synergism With Paclitaxel In Vitro
Source: Cancer Med. 2025 Nov 12;14(21):e71376. doi: 10.1002/cam4.71376 (PMC12611308; doi:10.1002/cam4.71376)
Supplement: Supplementary file 3 — Table S1: Genes upregulated (log2FC > 0.6) and downregulated (log2FC < −0.6) in the pCR S1R‐high versus RD S1R‐low group (GSE25066). [file CAM4-14-e71376-s004.docx]

**Supplementary Table 1:**

Genes upregulated (log_2_FC > 0.6) and downregulated (log_2_FC < -0.6) in the pCR*_S1R_*_-high_ vs RD*_S1R_*_-low_ group (GSE25066)

| **Downregulated genes** | |
| --- | --- |
| **ID** | **Gene Symbol** |
| 209460_at | ABAT |
| 209459_s_at | ABAT |
| 215465_at | ABCA12 |
| 208161_s_at | ABCC3 |
| 210246_s_at | ABCC8 |
| 204567_s_at | ABCG1 |
| 205730_s_at | ABLIM3 |
| 221928_at | ACACB |
| 205355_at | ACADSB |
| 212977_at | ACKR3 |
| 205364_at | ACOX2 |
| 218844_at | ACSF2 |
| 213245_at | ADCY1 |
| 204497_at | ADCY9 |
| 204966_at | ADGRB2 |
| 209613_s_at | ADH1B |
| 209612_s_at | ADH1B |
| 209869_at | ADRA2A |
| 201792_at | AEBP1 |
| 201924_at | AFF1 |
| 205734_s_at | AFF3 |
| 208016_s_at | AGTR1 |
| 205357_s_at | AGTR1 |
| 210517_s_at | AKAP12 |
| 216381_x_at | AKR7A3 |
| 206469_x_at | AKR7A3 |
| 201952_at | ALCAM |
| 201951_at | ALCAM |
| 204942_s_at | ALDH3B2 |
| 206714_at | ALOX15B |
| 222108_at | AMIGO2 |
| 218804_at | ANO1 |
| 210085_s_at | ANXA9 |
| 212985_at | APBB2 |
| 40148_at | APBB2 |
| 213419_at | APBB2 |
| 201525_at | APOD |
| 212583_at | AQR |
| 211110_s_at | AR |
| 205239_at | AREG |
| 201288_at | ARHGDIB |
| 218195_at | ARMT1 |
| 202986_at | ARNT2 |
| 210129_s_at | ARPC4-TTLL3///TTLL3///ARPC4 |
| 206831_s_at | ARSD |
| 213702_x_at | ASAH1 |
| 210980_s_at | ASAH1 |
| 209987_s_at | ASCL1 |
| 207522_s_at | ATP2A3 |
| 213036_x_at | ATP2A3 |
| 219659_at | ATP8A2 |
| 213745_at | ATRNL1 |
| 212745_s_at | BBS4 |
| 204378_at | BCAS1 |
| 203684_s_at | BCL2 |
| 203685_at | BCL2 |
| 218332_at | BEX1 |
| 201169_s_at | BHLHE40 |
| 205780_at | BIK |
| 203771_s_at | BLVRA |
| 202201_at | BLVRB |
| 210523_at | BMPR1B |
| 221479_s_at | BNIP3L |
| 201235_s_at | BTG2 |
| 201236_s_at | BTG2 |
| 220141_at | C11orf63 |
| 218820_at | C14orf132 |
| 212736_at | C16orf45 |
| 221272_s_at | C1orf21 |
| 221823_at | C5orf30 |
| 202992_at | C7 |
| 218541_s_at | C8orf4 |
| 210108_at | CACNA1D |
| 221585_at | CACNG4 |
| 218309_at | CAMK2N1 |
| 208063_s_at | CAPN9 |
| 212914_at | CBX7 |
| 220581_at | CCDC170 |
| 205392_s_at | CCL15-CCL14///CCL14 |
| 202769_at | CCNG2 |
| 211559_s_at | CCNG2 |
| 202770_s_at | CCNG2 |
| 221156_x_at | CCPG1 |
| 206488_s_at | CD36 |
| 208783_s_at | CD46 |
| 207173_x_at | CDH11 |
| 201884_at | CEACAM5 |
| 211657_at | CEACAM6 |
| 203757_s_at | CEACAM6 |
| 212442_s_at | CERS6 |
| 212446_s_at | CERS6 |
| 219455_at | CFAP69 |
| 202357_s_at | CFB |
| 205382_s_at | CFD |
| 206869_at | CHAD |
| 210075_at | 45353 |
| 211248_s_at | CHRD |
| 200811_at | CIRBP |
| 200810_s_at | CIRBP |
| 205830_at | CLGN |
| 204085_s_at | CLN5 |
| 219414_at | CLSTN2 |
| 215145_s_at | CNTNAP2 |
| 219301_s_at | CNTNAP2 |
| 202311_s_at | COL1A1 |
| 202310_s_at | COL1A1 |
| 202403_s_at | COL1A2 |
| 202404_s_at | COL1A2 |
| 201852_x_at | COL3A1 |
| 211161_s_at | COL3A1 |
| 215076_s_at | COL3A1 |
| 219625_s_at | COL4A3BP |
| 213110_s_at | COL4A5 |
| 212489_at | COL5A1 |
| 203325_s_at | COL5A1 |
| 221729_at | COL5A2 |
| 221730_at | COL5A2 |
| 201438_at | COL6A3 |
| 218439_s_at | COMMD10 |
| 205713_s_at | COMP |
| 204570_at | COX7A1 |
| 205624_at | CPA3 |
| 205509_at | CPB1 |
| 201940_at | CPD |
| 201117_s_at | CPE |
| 201116_s_at | CPE |
| 218610_s_at | CPPED1 |
| 205843_x_at | CRAT |
| 209522_s_at | CRAT |
| 213059_at | CREB3L1 |
| 205081_at | CRIP1 |
| 204573_at | CROT |
| 203591_s_at | CSF3R |
| 206224_at | CST1 |
| 202450_s_at | CTSK |
| 205898_at | CX3CR1 |
| 209687_at | CXCL12 |
| 218002_s_at | CXCL14 |
| 207094_at | CXCR1 |
| 215726_s_at | CYB5A |
| 207843_x_at | CYB5A |
| 209366_x_at | CYB5A |
| 217889_s_at | CYBRD1 |
| 215785_s_at | CYFIP2 |
| 207244_x_at | CYP2A6 |
| 210272_at | CYP2B7P |
| 210096_at | CYP4B1 |
| 205471_s_at | DACH1 |
| 205472_s_at | DACH1 |
| 212595_s_at | DAZAP2 |
| 200794_x_at | DAZAP2 |
| 205399_at | DCLK1 |
| 209335_at | DCN |
| 201893_x_at | DCN |
| 211813_x_at | DCN |
| 211896_s_at | DCN |
| 214079_at | DHRS2 |
| 206463_s_at | DHRS2 |
| 222068_s_at | DNAAF1 |
| 218976_at | DNAJC12 |
| 205186_at | DNALI1 |
| 213071_at | DPT |
| 213068_at | DPT |
| 201041_s_at | DUSP1 |
| 204015_s_at | DUSP4 |
| 204014_at | DUSP4 |
| 209457_at | DUSP5 |
| 208893_s_at | DUSP6 |
| 218025_s_at | ECI2 |
| 219833_s_at | EFHC1 |
| 45297_at | EHD2 |
| 220029_at | ELOVL2 |
| 213712_at | ELOVL2 |
| 208788_at | ELOVL5 |
| 205065_at | ENPP1 |
| 205066_s_at | ENPP1 |
| 206710_s_at | EPB41L3 |
| 37986_at | EPOR |
| 91826_at | EPS8L1 |
| 217190_x_at | ESR1 |
| 211234_x_at | ESR1 |
| 211233_x_at | ESR1 |
| 215552_s_at | ESR1 |
| 220134_x_at | EVA1B |
| 221710_x_at | EVA1B |
| 217838_s_at | EVL |
| 203980_at | FABP4 |
| 221959_at | FAM110B |
| 222085_at | FAM174B |
| 219872_at | FAM198B |
| 218390_s_at | FAM204A |
| 209955_s_at | FAP |
| 220751_s_at | FAXDC2 |
| 209696_at | FBP1 |
| 209005_at | FBXL5 |
| 209004_s_at | FBXL5 |
| 204007_at | FCGR3B |
| 218831_s_at | FCGRT |
| 204379_s_at | FGFR3 |
| 206857_s_at | FKBP1B |
| 219250_s_at | FLRT3 |
| 215300_s_at | FMO5 |
| 205776_at | FMO5 |
| 202709_at | FMOD |
| 204072_s_at | FRY |
| 214211_at | FTH1 |
| 203988_s_at | FUT8 |
| 221759_at | G6PC3 |
| 212256_at | GALNT10 |
| 207357_s_at | GALNT10 |
| 218313_s_at | GALNT7 |
| 205354_at | GAMT |
| 202191_s_at | GAS7 |
| 209710_at | GATA2 |
| 221577_x_at | GDF15 |
| 205696_s_at | GFRA1 |
| 201667_at | GJA1 |
| 206646_at | GLI1 |
| 207928_s_at | GLRA3 |
| 200947_s_at | GLUD1 |
| 200648_s_at | GLUL |
| 217202_s_at | GLUL |
| 204115_at | GNG11 |
| 212294_at | GNG12 |
| 202756_s_at | GPC1 |
| 212510_at | GPD1L |
| 219327_s_at | GPRC5C |
| 220340_at | GREB1L |
| 206326_at | GRP |
| 205439_at | GSTT2 |
| 215043_s_at | GUSBP3///SMA5///SMA4 |
| 213515_x_at | HBG2///HBG1 |
| 204419_x_at | HBG2///HBG1 |
| 202815_s_at | HEXIM1 |
| 214307_at | HGD |
| 205221_at | HGD |
| 204607_at | HMGCS2 |
| 211732_x_at | HNMT |
| 204112_s_at | HNMT |
| 211597_s_at | HOPX |
| 205453_at | HOXB2 |
| 205366_s_at | HOXB6 |
| 211549_s_at | HPGD |
| 210013_at | HPX |
| 201413_at | HSD17B4 |
| 211538_s_at | HSPA2 |
| 207404_s_at | HTR1E |
| 201185_at | HTRA1 |
| 212223_at | IDS |
| 201315_x_at | IFITM2 |
| 209540_at | IGF1 |
| 203627_at | IGF1R |
| 203628_at | IGF1R |
| 202718_at | IGFBP2 |
| 203424_s_at | IGFBP5 |
| 209341_s_at | IKBKB |
| 211027_s_at | IKBKB |
| 219115_s_at | IL20RA |
| 207538_at | IL4 |
| 204864_s_at | IL6ST |
| 211000_s_at | IL6ST |
| 204863_s_at | IL6ST |
| 212196_at | IL6ST |
| 212195_at | IL6ST |
| 205376_at | INPP4B |
| 213651_at | INPP5J |
| 202409_at | INS-IGF2///IGF2 |
| 203474_at | IQGAP2 |
| 204686_at | IRS1 |
| 201124_at | ITGB5 |
| 214021_x_at | ITGB5 |
| 214020_x_at | ITGB5 |
| 201125_s_at | ITGB5 |
| 217731_s_at | ITM2B |
| 216944_s_at | ITPR1 |
| 203710_at | ITPR1 |
| 211323_s_at | ITPR1 |
| 216958_s_at | IVD |
| 222256_s_at | JMJD7///JMJD7-PLA2G4B |
| 212462_at | KAT6B |
| 213832_at | KCND3 |
| 215014_at | KCND3 |
| 221583_s_at | KCNMA1 |
| 212492_s_at | KDM4B |
| 212496_s_at | KDM4B |
| 215616_s_at | KDM4B |
| 221874_at | KIAA1324 |
| 203130_s_at | KIF5C |
| 211124_s_at | KITLG |
| 219371_s_at | KLF2 |
| 203726_s_at | LAMA3 |
| 219463_at | LAMP5 |
| 204414_at | LARGE1 |
| 221558_s_at | LEF1 |
| 208933_s_at | LGALS8 |
| 212327_at | LIMCH1 |
| 219135_s_at | LMF1 |
| 209616_s_at | LOC100653057///CES1 |
| 221974_at | LOC101930404///SNORD116-28///SNORD115-26///SNORD115-13///SNORD115-7///SNORD116-22///SNORD116-4///PWARSN///SNORD107///SNRPN///IPW |
| 214203_s_at | LOC102724788///PRODH |
| 204674_at | LRMP |
| 213909_at | LRRC15 |
| 205381_at | LRRC17 |
| 220622_at | LRRC31 |
| 201744_s_at | LUM |
| 204952_at | LYPD3 |
| 213627_at | MAGED2 |
| 208682_s_at | MAGED2 |
| 218918_at | MAN1C1 |
| 212741_at | MAOA |
| 204041_at | MAOB |
| 220145_at | MAP9 |
| 212497_at | MAPK1IP1L |
| 203930_s_at | MAPT |
| 203929_s_at | MAPT |
| 203928_x_at | MAPT |
| 206401_s_at | MAPT |
| 210958_s_at | MAST4 |
| 206091_at | MATN3 |
| 209624_s_at | MCCC2 |
| 210794_s_at | MEG3 |
| 212830_at | MEGF9 |
| 214269_at | MFSD7 |
| 206522_at | MGAM |
| 219332_at | MICALL2 |
| 203071_at | MIR6872///SEMA3B |
| 202392_s_at | MIR7109///PISD |
| 203435_s_at | MME |
| 203434_s_at | MME |
| 219648_at | MREG |
| 210297_s_at | MSMB |
| 217977_at | MSRB1 |
| 210319_x_at | MSX2 |
| 205555_s_at | MSX2 |
| 207847_s_at | MUC1 |
| 206877_at | MXD1 |
| 210480_s_at | MYO6 |
| 204139_x_at | MZF1 |
| 210336_x_at | MZF1 |
| 204861_s_at | NAIP |
| 204860_s_at | NAIP |
| 214440_at | NAT1 |
| 209930_s_at | NFE2 |
| 219438_at | NKAIN1 |
| 204862_s_at | NME3 |
| 206197_at | NME5 |
| 205794_s_at | NOVA1 |
| 218086_at | NPDC1 |
| 201468_s_at | NQO1 |
| 209505_at | NR2F1 |
| 204081_at | NRGN |
| 202600_s_at | NRIP1 |
| 202599_s_at | NRIP1 |
| 221796_at | NTRK2 |
| 206291_at | NTS |
| 212183_at | NUDT4P1///NUDT4 |
| 218730_s_at | OGN |
| 213131_at | OLFM1 |
| 205591_at | OLFM1 |
| 220005_at | P2RY13 |
| 206228_at | PAX2 |
| 202465_at | PCOLCE |
| 205872_x_at | PDE4DIP |
| 202273_at | PDGFRB |
| 205380_at | PDZK1 |
| 205160_at | PEX11A |
| 207132_x_at | PFDN5 |
| 208305_at | PGR |
| 221756_at | PIK3IP1 |
| 206509_at | PIP |
| 201133_s_at | PJA2 |
| 209581_at | PLA2G16 |
| 201860_s_at | PLAT |
| 218640_s_at | PLEKHF2 |
| 201939_at | PLK2 |
| 220798_x_at | PLPPR3 |
| 206470_at | PLXNC1 |
| 210139_s_at | PMP22 |
| 219756_s_at | POF1B |
| 202996_at | POLD4 |
| 210809_s_at | POSTN |
| 214146_s_at | PPBP |
| 210832_x_at | PTGER3 |
| 213933_at | PTGER3 |
| 213691_at | PTOV1-AS2 |
| 203030_s_at | PTPRN2 |
| 203029_s_at | PTPRN2 |
| 205948_at | PTPRT |
| 205336_at | PVALB |
| 221666_s_at | PYCARD |
| 209123_at | QDPR |
| 219681_s_at | RAB11FIP1 |
| 219562_at | RAB26 |
| 207018_s_at | RAB27B |
| 214552_s_at | RABEP1 |
| 219440_at | RAI2 |
| 211605_s_at | RARA |
| 203749_s_at | RARA |
| 204070_at | RARRES3 |
| 218035_s_at | RBM47 |
| 204365_s_at | REEP1 |
| 204364_s_at | REEP1 |
| 205645_at | REPS2 |
| 211421_s_at | RET |
| 206107_at | RGS11 |
| 218353_at | RGS5 |
| 209070_s_at | RGS5 |
| 209071_s_at | RGS5 |
| 218686_s_at | RHBDF1 |
| 204951_at | RHOH |
| 213397_x_at | RNASE4 |
| 205158_at | RNASE4 |
| 210056_at | RND1 |
| 202636_at | RNF103 |
| 218394_at | ROGDI |
| 210222_s_at | RTN1 |
| 203485_at | RTN1 |
| 218677_at | S100A14 |
| 201826_s_at | SCCPDH |
| 206799_at | SCGB1D2 |
| 205979_at | SCGB2A1 |
| 206378_at | SCGB2A2 |
| 219197_s_at | SCUBE2 |
| 204541_at | SEC14L2 |
| 203788_s_at | SEMA3C |
| 203789_s_at | SEMA3C |
| 206941_x_at | SEMA3E |
| 201427_s_at | SEPP1 |
| 217284_x_at | SERHL2 |
| 217276_x_at | SERHL2 |
| 214243_s_at | SERHL2///SERHL |
| 211429_s_at | SERPINA1 |
| 202833_s_at | SERPINA1 |
| 205352_at | SERPINI1 |
| 201311_s_at | SH3BGRL |
| 201312_s_at | SH3BGRL |
| 222258_s_at | SH3BP4 |
| 218813_s_at | SH3GLB2 |
| 213464_at | SHC2 |
| 219734_at | SIDT1 |
| 220503_at | SLC13A1 |
| 205234_at | SLC16A4 |
| 207038_at | SLC16A6 |
| 206396_at | SLC1A1 |
| 213664_at | SLC1A1 |
| 204981_at | SLC22A18 |
| 206081_at | SLC24A1 |
| 206143_at | SLC26A3 |
| 205768_s_at | SLC27A2 |
| 214719_at | SLC46A3 |
| 218417_s_at | SLC48A1 |
| 207056_s_at | SLC4A8 |
| 207626_s_at | SLC7A2 |
| 216604_s_at | SLC7A8 |
| 216603_at | SLC7A8 |
| 209453_at | SLC9A1 |
| 214850_at | SMA4 |
| 206565_x_at | SMA4 |
| 209131_s_at | SNAP23 |
| 204466_s_at | SNCA |
| 201522_x_at | SNURF///SNRPN |
| 206042_x_at | SNURF///SNRPN |
| 218087_s_at | SORBS1 |
| 217575_s_at | SOS2 |
| 212667_at | SPARC |
| 213441_x_at | SPDEF |
| 220192_x_at | SPDEF |
| 214404_x_at | SPDEF |
| 212458_at | SPRED2 |
| 219919_s_at | SSH3 |
| 204542_at | ST6GALNAC2 |
| 213103_at | STARD13 |
| 203439_s_at | STC2 |
| 220187_at | STEAP4 |
| 219686_at | STK32B |
| 212354_at | SULF1 |
| 212353_at | SULF1 |
| 212344_at | SULF1 |
| 205342_s_at | SULT1C2 |
| 203998_s_at | SYT1 |
| 205613_at | SYT17 |
| 220613_s_at | SYTL2 |
| 211144_x_at | TARP///TRGV9///TRGC2 |
| 209813_x_at | TARP///TRGV9///TRGC2 |
| 206916_x_at | TAT |
| 219682_s_at | TBX3 |
| 202371_at | TCEAL4 |
| 205513_at | TCN1 |
| 215686_x_at | TFAP2B |
| 214451_at | TFAP2B |
| 205009_at | TFF1 |
| 209278_s_at | TFPI2 |
| 201107_s_at | THBS1 |
| 211155_s_at | THPO |
| 201666_at | TIMP1 |
| 212770_at | TLE3 |
| 219206_x_at | TMBIM4 |
| 219580_s_at | TMC5 |
| 219663_s_at | TMEM121 |
| 217613_at | TMEM144 |
| 206222_at | TNFRSF10C |
| 211163_s_at | TNFRSF10C |
| 210654_at | TNFRSF10D |
| 211828_s_at | TNIK |
| 213201_s_at | TNNT1 |
| 215108_x_at | TOX3 |
| 214774_x_at | TOX3 |
| 216623_x_at | TOX3 |
| 210886_x_at | TP53TG1 |
| 209917_s_at | TP53TG1 |
| 210241_s_at | TP53TG1 |
| 207741_x_at | TPSAB1 |
| 215382_x_at | TPSAB1 |
| 216474_x_at | TPSB2///TPSAB1 |
| 217023_x_at | TPSB2///TPSAB1 |
| 204079_at | TPST2 |
| 210995_s_at | TRIM23 |
| 215047_at | TRIM58 |
| 221012_s_at | TRIM8 |
| 208763_s_at | TSC22D3 |
| 217979_at | TSPAN13 |
| 203227_s_at | TSPAN31 |
| 210652_s_at | TTC39A |
| 201534_s_at | UBL3 |
| 221765_at | UGCG |
| 203343_at | UGDH |
| 211682_x_at | UGT2B28 |
| 218807_at | VAV3 |
| 218806_s_at | VAV3 |
| 219609_at | WDR25 |
| 221958_s_at | WLS |
| 208606_s_at | WNT4 |
| 212637_s_at | WWP1 |
| 221728_x_at | XIST |
| 210709_at | ZC3H15 |
| 212419_at | ZCCHC24 |
| 212423_at | ZCCHC24 |
| 212764_at | ZEB1 |
| 205714_s_at | ZMYND10 |
| 219900_s_at | ZNF446 |
| 219741_x_at | ZNF552 |
| 215570_s_at | ZNF780A///ZNF780B |
| 213605_s_at | |
| 217363_x_at | |
| 216494_at | |
| 220296_at | |
| 207470_at | |
| 217572_at | |

| **Upregulated Genes** | |
| --- | --- |
| **Gene ID** | **Gene Symbol** |
| 218405_at | ABT1 |
| 201629_s_at | ACP1 |
| 214957_at | ACTL8 |
| 218868_at | ACTR3B |
| 217007_s_at | ADAM15 |
| 206002_at | ADGRG2 |
| 213094_at | ADGRG6 |
| 206953_s_at | ADGRL2 |
| 218480_at | AGBL5 |
| 219693_at | AGPAT4 |
| 206957_at | AGXT |
| 212173_at | AK2 |
| 205706_s_at | ANKRD26 |
| 209870_s_at | APBA2 |
| 214960_at | API5 |
| 206632_s_at | APOBEC3B |
| 203263_s_at | ARHGEF9 |
| 203264_s_at | ARHGEF9 |
| 214943_s_at | ARID4B///RBM34 |
| 220658_s_at | ARNTL2 |
| 210147_at | ART3 |
| 205047_s_at | ASNS |
| 219918_s_at | ASPM |
| 207076_s_at | ASS1 |
| 218782_s_at | ATAD2 |
| 215842_s_at | ATP11A |
| 213582_at | ATP11A |
| 205704_s_at | ATP6V0A2 |
| 209902_at | ATR |
| 208079_s_at | AURKA |
| 204092_s_at | AURKA |
| 209464_at | AURKB |
| 219366_at | AVEN |
| 217867_x_at | BACE2 |
| 209406_at | BAG2 |
| 205345_at | BARD1 |
| 205363_at | BBOX1 |
| 210347_s_at | BCL11A |
| 202095_s_at | BIRC5 |
| 202094_at | BIRC5 |
| 221703_at | BRIP1 |
| 219177_at | BRIX1 |
| 202946_s_at | BTBD3 |
| 205548_s_at | BTG3 |
| 209642_at | BUB1 |
| 203755_at | BUB1B |
| 203612_at | BYSL |
| 219785_s_at | C16orf95 |
| 219010_at | C1orf106 |
| 220840_s_at | C1orf112 |
| 217924_at | C6orf106 |
| 222309_at | C6orf62 |
| 205199_at | CA9 |
| 210691_s_at | CACYBP |
| 201381_x_at | CACYBP |
| 202715_at | CAD |
| 205428_s_at | CALB2 |
| 201616_s_at | CALD1 |
| 201615_x_at | CALD1 |
| 212077_at | CALD1 |
| 215198_s_at | CALD1 |
| 221879_at | CALML4 |
| 64408_s_at | CALML4 |
| 220414_at | CALML5 |
| 200755_s_at | CALU |
| 202965_s_at | CAPN6 |
| 213310_at | CASC7///AGO2 |
| 209213_at | CBR1 |
| 205379_at | CBR3 |
| 212816_s_at | CBS |
| 219387_at | CCDC88A |
| 209698_at | CCHCR1 |
| 37425_g_at | CCHCR1 |
| 209924_at | CCL18 |
| 216598_s_at | CCL2 |
| 214038_at | CCL8 |
| 213226_at | CCNA2 |
| 203418_at | CCNA2 |
| 214710_s_at | CCNB1 |
| 217988_at | CCNB1IP1 |
| 202705_at | CCNB2 |
| 205034_at | CCNE2 |
| 204827_s_at | CCNF |
| 201327_s_at | CCT6A |
| 208650_s_at | CD24 |
| 218529_at | CD320 |
| 202870_s_at | CDC20 |
| 205167_s_at | CDC25C |
| 204693_at | CDC42EP1 |
| 203967_at | CDC6 |
| 221436_s_at | CDCA3 |
| 203256_at | CDH3 |
| 203213_at | CDK1 |
| 210559_s_at | CDK1 |
| 203214_x_at | CDK1 |
| 201938_at | CDK2AP1 |
| 220935_s_at | CDK5RAP2 |
| 207143_at | CDK6 |
| 211792_s_at | CDKN2C |
| 204159_at | CDKN2C |
| 209832_s_at | CDT1 |
| 212501_at | CEBPB |
| 204203_at | CEBPG |
| 205046_at | CENPE |
| 207828_s_at | CENPF |
| 209172_s_at | CENPF |
| 214804_at | CENPI |
| 207590_s_at | CENPI |
| 218741_at | CENPM |
| 219555_s_at | CENPN |
| 222118_at | CENPN |
| 218883_s_at | CENPU |
| 214742_at | CEP131 |
| 212746_s_at | CEP170P1///CEP170 |
| 218542_at | CEP55 |
| 219311_at | CEP76 |
| 52285_f_at | CEP76 |
| 203976_s_at | CHAF1A |
| 214426_x_at | CHAF1A |
| 217972_at | CHCHD3 |
| 205393_s_at | CHEK1 |
| 205394_at | CHEK1 |
| 209395_at | CHI3L1 |
| 209396_s_at | CHI3L1 |
| 213060_s_at | CHI3L2 |
| 219867_at | CHODL |
| 214596_at | CHRM3 |
| 209834_at | CHST3 |
| 205008_s_at | CIB2 |
| 205516_x_at | CIZ1 |
| 218252_at | CKAP2 |
| 201897_s_at | CKS1B |
| 204170_s_at | CKS2 |
| 214769_at | CLCN4 |
| 201560_at | CLIC4 |
| 221881_s_at | CLIC4 |
| 218161_s_at | CLN6 |
| 218447_at | CMC2 |
| 219420_s_at | COA7 |
| 205229_s_at | COCH |
| 211966_at | COL4A2 |
| 204724_s_at | COL9A3 |
| 213736_at | COX5B |
| 205350_at | CRABP1 |
| 209283_at | CRYAB |
| 210766_s_at | CSE1L |
| 201111_at | CSE1L |
| 207803_s_at | CSN3 |
| 211126_s_at | CSRP2 |
| 210546_x_at | CTAG1A///CTAG1B |
| 201487_at | CTSC |
| 210074_at | CTSV |
| 203687_at | CX3CL1 |
| 823_at | CX3CL1 |
| 203917_at | CXADR |
| 204533_at | CXCL10 |
| 210163_at | CXCL11 |
| 211122_s_at | CXCL11 |
| 205242_at | CXCL13 |
| 209774_x_at | CXCL2 |
| 203915_at | CXCL9 |
| 211469_s_at | CXCR6 |
| 215121_x_at | CYAT1///IGLV1-44///IGLC1 |
| 220230_s_at | CYB5R2 |
| 220432_s_at | CYP39A1 |
| 204244_s_at | DBF4 |
| 221849_s_at | DCAF15 |
| 222233_s_at | DCLRE1C |
| 218774_at | DCPS |
| 204851_s_at | DCX |
| 202887_s_at | DDIT4 |
| 210206_s_at | DDX11 |
| 208896_at | DDX18 |
| 203785_s_at | DDX28 |
| 218943_s_at | DDX58 |
| 210397_at | DEFB1 |
| 220295_x_at | DEPDC1 |
| 212371_at | DESI2 |
| 206752_s_at | DFFB |
| 201791_s_at | DHCR7 |
| 201790_s_at | DHCR7 |
| 218756_s_at | DHRS11 |
| 209916_at | DHTKD1 |
| 205603_s_at | DIAPH2 |
| 211150_s_at | DLAT |
| 203764_at | DLGAP5 |
| 203881_s_at | DMD |
| 220668_s_at | DNMT3B |
| 204238_s_at | DNPH1 |
| 204751_x_at | DSC2 |
| 219000_s_at | DSCC1 |
| 217901_at | DSG2 |
| 218585_s_at | DTL |
| 214253_s_at | DTNB |
| 204794_at | DUSP2 |
| 38157_at | DXO |
| 204557_s_at | DZIP1 |
| 204947_at | E2F1 |
| 221586_s_at | E2F5 |
| 219990_at | E2F8 |
| 213789_at | EBP |
| 202735_at | EBP |
| 219787_s_at | ECT2 |
| 210132_at | EFNA3 |
| 205107_s_at | EFNA4 |
| 201983_s_at | EGFR |
| 208427_s_at | ELAVL2 |
| 212670_at | ELN |
| 212396_s_at | EMC1 |
| 220559_at | EN1 |
| 217294_s_at | ENO1 |
| 201718_s_at | EPB41L2 |
| 201839_s_at | EPCAM |
| 1438_at | EPHB3 |
| 219650_at | ERCC6L |
| 38158_at | ESPL1 |
| 204603_at | EXO1 |
| 214507_s_at | EXOSC2 |
| 218695_at | EXOSC4 |
| 209692_at | EYA2 |
| 203358_s_at | EZH2 |
| 202345_s_at | FABP5 |
| 205029_s_at | FABP7 |
| 205030_at | FABP7 |
| 202218_s_at | FADS2 |
| 218080_x_at | FAF1 |
| 209074_s_at | FAM107A |
| 212771_at | FAM171A1 |
| 203550_s_at | FAM189B |
| 218331_s_at | FAM208B |
| 209683_at | FAM49A |
| 221591_s_at | FAM64A |
| 203806_s_at | FANCA |
| 203805_s_at | FANCA |
| 220255_at | FANCE |
| 203564_at | FANCG |
| 213008_at | FANCI |
| 213007_at | FANCI |
| 201911_s_at | FARP1 |
| 216602_s_at | FARSA |
| 201579_at | FAT1 |
| 219208_at | FBXO11 |
| 218875_s_at | FBXO5 |
| 201275_at | FDPS |
| 218796_at | FERMT1 |
| 60474_at | FERMT1 |
| 219187_at | FKBPL |
| 219316_s_at | FLVCR2 |
| 211303_x_at | FOLH1B |
| 205860_x_at | FOLH1B///FOLH1 |
| 215363_x_at | FOLH1B///FOLH1 |
| 206307_s_at | FOXD1 |
| 202580_x_at | FOXM1 |
| 208475_at | FRMD4A |
| 208476_s_at | FRMD4A |
| 201564_s_at | FSCN1 |
| 210933_s_at | FSCN1 |
| 203706_s_at | FZD7 |
| 203705_s_at | FZD7 |
| 214240_at | GAL |
| 203397_s_at | GALNT3 |
| 217445_s_at | GART |
| 202270_at | GBP1 |
| 202269_x_at | GBP1 |
| 210175_at | GCFC2 |
| 211020_at | GCNT2 |
| 205527_s_at | GEMIN4 |
| 203560_at | GGH |
| 206102_at | GINS1 |
| 221521_s_at | GINS2 |
| 218719_s_at | GINS3 |
| 45633_at | GINS3 |
| 211767_at | GINS4 |
| 204836_at | GLDC |
| 204875_s_at | GMDS |
| 209169_at | GPM6B |
| 206972_s_at | GPR161 |
| 222140_s_at | GPR89A///GPR89B |
| 203632_s_at | GPRC5B |
| 205240_at | GPSM2 |
| 221922_at | GPSM2 |
| 213170_at | GPX7 |
| 200824_at | GSTP1 |
| 215942_s_at | GTSE1 |
| 204315_s_at | GTSE1 |
| 204318_s_at | GTSE1 |
| 205436_s_at | H2AFX |
| 218445_at | H2AFY2 |
| 200853_at | H2AFZ |
| 219654_at | HACD1 |
| 218602_s_at | HAUS6 |
| 201833_at | HDAC2 |
| 200896_x_at | HDGF |
| 220085_at | HELLS |
| 219352_at | HERC6 |
| 212964_at | HIC2 |
| 206495_s_at | HINFP |
| 218726_at | HJURP |
| 205671_s_at | HLA-DOB |
| 206074_s_at | HMGA1 |
| 207165_at | HMMR |
| 217755_at | HN1 |
| 213793_s_at | HOMER1 |
| 204647_at | HOMER3 |
| 210112_at | HPS1 |
| 219983_at | HRASLS |
| 205829_at | HSD17B1 |
| 204818_at | HSD17B2 |
| 219212_at | HSPA14 |
| 205543_at | HSPA4L |
| 202601_s_at | HTATSF1 |
| 203089_s_at | HTRA2 |
| 209291_at | ID4 |
| 209292_at | ID4 |
| 210046_s_at | IDH2 |
| 210029_at | IDO1 |
| 214453_s_at | IFI44 |
| 204439_at | IFI44L |
| 219209_at | IFIH1 |
| 202147_s_at | IFRD1 |
| 202146_at | IFRD1 |
| 219174_at | IFT74 |
| 203819_s_at | IGF2BP3 |
| 216491_x_at | IGHM |
| 209374_s_at | IGHM |
| 215949_x_at | IGHM |
| 216510_x_at | IGHV3-23///IGHV4-31///IGHM///IGHG1///IGHA1 |
| 211650_x_at | IGHV3-23///IGHV4-31///IGHM///IGHG3///IGHG1///IGHD///IGHA1///IGH |
| 211637_x_at | IGHV3-23///IGHV4-31///IGHM///IGHG4///IGHG3///IGHG1///IGHD///IGHA2///IGHA1///IGH |
| 217360_x_at | IGHV4-31///IGHM///IGHG3///IGHG1///IGHA1 |
| 216557_x_at | IGHV4-31///IGHM///IGHG3///IGHG1///IGHD///IGHA1 |
| 211868_x_at | IGHV4-31///IGHM///IGHG3///IGHG2///IGHG1///IGHD///IGHA2///IGHA1///IGH |
| 211639_x_at | IGHV4-31///IGHM///IGHG4///IGHG3///IGHG1///IGHD///IGHA2///IGHA1///IGH |
| 211908_x_at | IGK |
| 215176_x_at | IGK///IGKC |
| 214777_at | IGKC |
| 217378_x_at | IGKV1OR2-108 |
| 216560_x_at | IGLC1 |
| 209138_x_at | IGLC1 |
| 214677_x_at | IGLC1 |
| 211798_x_at | IGLJ3 |
| 216853_x_at | IGLJ3 |
| 216365_x_at | IGLJ3///CKAP2 |
| 215946_x_at | IGLL3P |
| 215379_x_at | IGLV1-44 |
| 217258_x_at | IGLV1-44 |
| 222062_at | IL27RA |
| 203126_at | IMPA2 |
| 218516_s_at | IMPAD1 |
| 219769_at | INCENP |
| 210350_x_at | ING1 |
| 221185_s_at | IQCG |
| 201587_s_at | IRAK1 |
| 221004_s_at | ITM2C |
| 218560_s_at | JMJD4 |
| 206734_at | JRKL |
| 203163_at | KATNB1 |
| 213478_at | KAZN |
| 210078_s_at | KCNAB1 |
| 214595_at | KCNG1 |
| 219615_s_at | KCNK5 |
| 219479_at | KDELC1 |
| 212348_s_at | KDM1A |
| 209781_s_at | KHDRBS3 |
| 204444_at | KIF11 |
| 206364_at | KIF14 |
| 219306_at | KIF15 |
| 221258_s_at | KIF18A |
| 203849_s_at | KIF1A |
| 218755_at | KIF20A |
| 204709_s_at | KIF23 |
| 209408_at | KIF2C |
| 218355_at | KIF4A |
| 209680_s_at | KIFC1 |
| 205051_s_at | KIT |
| 221986_s_at | KLHL24 |
| 219453_at | KLHL36 |
| 220682_s_at | KLHL5 |
| 220238_s_at | KLHL7 |
| 220239_at | KLHL7 |
| 222242_s_at | KLK5 |
| 205778_at | KLK7 |
| 206316_s_at | KNTC1 |
| 201088_at | KPNA2 |
| 211762_s_at | KPNA2 |
| 209351_at | KRT14 |
| 209800_at | KRT16 |
| 205157_s_at | KRT17///JUP |
| 212236_x_at | KRT17///JUP |
| 218963_s_at | KRT23 |
| 201820_at | KRT5 |
| 213680_at | KRT6B |
| 209126_x_at | KRT6B |
| 216641_s_at | LAD1 |
| 206486_at | LAG3 |
| 207816_at | LALBA |
| 205569_at | LAMP3 |
| 208029_s_at | LAPTM4B |
| 214039_s_at | LAPTM4B |
| 208767_s_at | LAPTM4B |
| 214216_s_at | LARP4B |
| 208117_s_at | LAS1L |
| 207022_s_at | LDHC |
| 219998_at | LGALSL |
| 212957_s_at | LINC01278 |
| 216952_s_at | LMNB2 |
| 216841_s_at | LOC100129518///SOD2 |
| 217384_x_at | LOC100293211 |
| 204347_at | LOC100507855///AK4 |
| 217281_x_at | LOC102725526///IGHV4-31///IGHM///IGHG3///IGHG2///IGHG1///IGHA2///IGHA1///IGH |
| 222031_at | LOC389906 |
| 208107_s_at | LOC81691 |
| 212276_at | LPIN1 |
| 219491_at | LRFN4 |
| 220253_s_at | LRP12 |
| 220254_at | LRP12 |
| 219631_at | LRP12 |
| 209468_at | LRP5 |
| 205606_at | LRP6 |
| 205282_at | LRP8 |
| 208433_s_at | LRP8 |
| 212529_at | LSM12 |
| 208190_s_at | LSR |
| 202728_s_at | LTBP1 |
| 206276_at | LY6D |
| 202145_at | LY6E |
| 203362_s_at | MAD2L1 |
| 218894_s_at | MAGOHB |
| 210093_s_at | MAGOHB///MAGOH |
| 209373_at | MALL |
| 208309_s_at | MALT1 |
| 206571_s_at | MAP4K4 |
| 211081_s_at | MAP4K5 |
| 219576_at | MAP7D3 |
| 205819_at | MARCO |
| 218440_at | MCCC1 |
| 220651_s_at | MCM10 |
| 212141_at | MCM4 |
| 222036_s_at | MCM4 |
| 222037_at | MCM4 |
| 212142_at | MCM4 |
| 201755_at | MCM5 |
| 208795_s_at | MCM7 |
| 210983_s_at | MCM7 |
| 220094_s_at | MCUR1 |
| 209035_at | MDK |
| 203496_s_at | MED1 |
| 203510_at | MET |
| 220007_at | METTL8 |
| 218247_s_at | MEX3C |
| 203417_at | MFAP2 |
| 210605_s_at | MFGE8 |
| 212945_s_at | MGA |
| 206560_s_at | MIA |
| 212715_s_at | MICAL3 |
| 221779_at | MICALL1 |
| 55081_at | MICALL1 |
| 203637_s_at | MID1 |
| 203636_at | MID1 |
| 213189_at | MINA |
| 209219_at | MIR1236///NELFE |
| 221580_s_at | MIR1304///SNORD5///SNORA32///SNORA40///SNORA18///SNORA1///SNORA8///TAF1D |
| 208963_x_at | MIR1908///FADS1 |
| 208962_s_at | MIR1908///FADS1 |
| 209825_s_at | MIR3658///UCK2 |
| 216212_s_at | MIR664B///SNORA56///DKC1 |
| 201478_s_at | MIR664B///SNORA56///DKC1 |
| 33132_at | MIR6849///MIR1234///MIR939///CPSF1 |
| 212563_at | MIR7112///BOP1 |
| 206500_s_at | MIS18BP1 |
| 212022_s_at | MKI67 |
| 212020_s_at | MKI67 |
| 212023_s_at | MKI67 |
| 204784_s_at | MLF1 |
| 216401_x_at | MLIP |
| 216503_s_at | MLLT10 |
| 204580_at | MMP12 |
| 204259_at | MMP7 |
| 203644_s_at | MON1B |
| 213306_at | MPDZ |
| 215731_s_at | MPHOSPH9 |
| 203780_at | MPZL2 |
| 203779_s_at | MPZL2 |
| 206538_at | MRAS |
| 205395_s_at | MRE11A |
| 203931_s_at | MRPL12 |
| 218027_at | MRPL15 |
| 211594_s_at | MRPL9 |
| 210008_s_at | MRPS12 |
| 221437_s_at | MRPS15 |
| 205122_at | MSANTD3-TMEFF1///TMEFF1 |
| 210410_s_at | MSH5-SAPCD1///SAPCD1///MSH5 |
| 211450_s_at | MSH6 |
| 204885_s_at | MSLN |
| 217546_at | MT1M |
| 216685_s_at | MTAP |
| 204956_at | MTAP |
| 219363_s_at | MTERF3 |
| 201761_at | MTHFD2 |
| 220196_at | MUC16 |
| 213432_at | MUC5B |
| 202086_at | MX1 |
| 213906_at | MYBL1 |
| 201710_at | MYBL2 |
| 202431_s_at | MYC |
| 201976_s_at | MYO10 |
| 201774_s_at | NCAPD2 |
| 218663_at | NCAPG |
| 218662_s_at | NCAPG |
| 212949_at | NCAPH |
| 218266_s_at | NCS1 |
| 204162_at | NDC80 |
| 200632_s_at | NDRG1 |
| 219006_at | NDUFAF4 |
| 219545_at | NDUFC2-KCTD14///KCTD14 |
| 204641_at | NEK2 |
| 211080_s_at | NEK2 |
| 218678_at | NES |
| 201830_s_at | NET1 |
| 218888_s_at | NETO2 |
| 211091_s_at | NF2 |
| 204702_s_at | NFE2L3 |
| 210268_at | NFX1 |
| 219031_s_at | NIP7 |
| 206023_at | NMU |
| 219324_at | NOL12 |
| 218199_s_at | NOL6 |
| 218051_s_at | NT5DC2 |
| 204766_s_at | NUDT1 |
| 206550_s_at | NUP155 |
| 212709_at | NUP160 |
| 212315_s_at | NUP210 |
| 202188_at | NUP93 |
| 219978_s_at | NUSAP1 |
| 218039_at | NUSAP1 |
| 219489_s_at | NXN |
| 212768_s_at | OLFM4 |
| 222304_x_at | OR7E47P |
| 205085_at | ORC1 |
| 219105_x_at | ORC6 |
| 210448_s_at | P2RX5 |
| 218744_s_at | PACSIN3 |
| 201014_s_at | PAICS |
| 218886_at | PAK1IP1 |
| 209043_at | PAPSS1 |
| 210094_s_at | PARD3 |
| 221526_x_at | PARD3 |
| 220060_s_at | PARPBP |
| 205646_s_at | PAX6 |
| 219148_at | PBK |
| 206935_at | PCDH8 |
| 216313_at | PCDHB17P |
| 201202_at | PCNA |
| 218952_at | PCSK1N |
| 217624_at | PDAP1 |
| 206348_s_at | PDK3 |
| 202671_s_at | PDXK |
| 217744_s_at | PERP |
| 202212_at | PES1 |
| 218336_at | PFDN2 |
| 201037_at | PFKP |
| 204992_s_at | PFN2 |
| 219225_at | PGBD5 |
| 207469_s_at | PIR |
| 208502_s_at | PITX1 |
| 209587_at | PITX1 |
| 221854_at | PKP1 |
| 210145_at | PLA2G4A |
| 207002_s_at | PLAGL1 |
| 209318_x_at | PLAGL1 |
| 203896_s_at | PLCB4 |
| 203895_at | PLCB4 |
| 219566_at | PLEKHF1 |
| 202240_at | PLK1 |
| 204886_at | PLK4 |
| 211088_s_at | PLK4 |
| 204887_s_at | PLK4 |
| 200827_at | PLOD1 |
| 202620_s_at | PLOD2 |
| 202619_s_at | PLOD2 |
| 201215_at | PLS3 |
| 202446_s_at | PLSCR1 |
| 202075_s_at | PLTP |
| 221538_s_at | PLXNA1 |
| 211013_x_at | PML |
| 206503_x_at | PML |
| 219152_at | PODXL2 |
| 218587_s_at | POGLUT1 |
| 204441_s_at | POLA2 |
| 203422_at | POLD1 |
| 209511_at | POLR2F |
| 213449_at | POP1 |
| 208928_at | POR |
| 208044_s_at | PPARD |
| 37152_at | PPARD |
| 204228_at | PPIH |
| 200913_at | PPM1G |
| 201407_s_at | PPP1CB |
| 212680_x_at | PPP1R14B |
| 205478_at | PPP1R1A |
| 219264_s_at | PPP2R3B |
| 218009_s_at | PRC1 |
| 201923_at | PRDX4 |
| 206273_at | PRELID3A |
| 205628_at | PRIM2 |
| 211084_x_at | PRKD3 |
| 208694_at | PRKDC |
| 204061_at | PRKX |
| 204060_s_at | PRKY///PRKX |
| 201300_s_at | PRNP |
| 204304_s_at | PROM1 |
| 204795_at | PRR3 |
| 211947_s_at | PRRC2C |
| 220126_at | PRSS50 |
| 220892_s_at | PSAT1 |
| 205961_s_at | PSIP1 |
| 201252_at | PSMC4 |
| 212219_at | PSME4 |
| 209815_at | PTCH1 |
| 207177_at | PTGFR |
| 204748_at | PTGS2 |
| 207011_s_at | PTK7 |
| 203038_at | PTPRK |
| 203554_x_at | PTTG1 |
| 208511_at | PTTG3P |
| 206157_at | PTX3 |
| 218670_at | PUS1 |
| 212662_at | PVR |
| 209336_at | PWP2 |
| 212265_at | QKI |
| 220955_x_at | RAB23 |
| 204547_at | RAB40B |
| 206103_at | RAC3 |
| 222077_s_at | RACGAP1 |
| 204146_at | RAD51AP1 |
| 204558_at | RAD54L |
| 202483_s_at | RANBP1 |
| 202482_x_at | RANBP1 |
| 213019_at | RANBP6 |
| 212127_at | RANGAP1 |
| 204681_s_at | RAPGEF5 |
| 206392_s_at | RARRES1 |
| 221872_at | RARRES1 |
| 219752_at | RASAL1 |
| 205296_at | RBL1 |
| 215089_s_at | RBM10 |
| 208984_x_at | RBM10 |
| 217221_x_at | RBM10 |
| 219286_s_at | RBM15 |
| 217857_s_at | RBM8A |
| 203423_at | RBP1 |
| 213520_at | RECQL4 |
| 218767_at | REXO4 |
| 1053_at | RFC2 |
| 218564_at | RFWD3 |
| 209544_at | RIPK2 |
| 221215_s_at | RIPK4 |
| 204669_s_at | RNF24 |
| 221909_at | RNFT2 |
| 202762_at | ROCK2 |
| 205805_s_at | ROR1 |
| 210115_at | RPL39L |
| 214097_at | RPS21 |
| 201203_s_at | RRBP1 |
| 201890_at | RRM2 |
| 209773_s_at | RRM2 |
| 218758_s_at | RRP1 |
| 212846_at | RRP1B |
| 202937_x_at | RRP7A |
| 204133_at | RRP9 |
| 201980_s_at | RSU1 |
| 201614_s_at | RUVBL1 |
| 205334_at | S100A1 |
| 214456_x_at | SAA2///SAA1 |
| 205449_at | SAC3D1 |
| 204900_x_at | SAP30 |
| 215834_x_at | SCARB1 |
| 205475_at | SCRG1 |
| 209631_s_at | SEL1L2///GPR37 |
| 208941_s_at | SEPHS1 |
| 204855_at | SERPINB5 |
| 212190_at | SERPINE2 |
| 207714_s_at | SERPINH1 |
| 209381_x_at | SF3A2 |
| 200687_s_at | SF3B3 |
| 202036_s_at | SFRP1 |
| 202035_s_at | SFRP1 |
| 202037_s_at | SFRP1 |
| 219493_at | SHCBP1 |
| 214095_at | SHMT2 |
| 214437_s_at | SHMT2 |
| 210792_x_at | SIVA1 |
| 206634_at | SIX3 |
| 217640_x_at | SKA1 |
| 210567_s_at | SKP2 |
| 219386_s_at | SLAMF8 |
| 204928_s_at | SLC10A3 |
| 220281_at | SLC12A1 |
| 219874_at | SLC12A8 |
| 209900_s_at | SLC16A1 |
| 202235_at | SLC16A1 |
| 202234_s_at | SLC16A1 |
| 204962_s_at | SLC35F6///CENPA |
| 210692_s_at | SLC43A3 |
| 222155_s_at | SLC52A2 |
| 204087_s_at | SLC5A6 |
| 219795_at | SLC6A14 |
| 201195_s_at | SLC7A5 |
| 214930_at | SLITRK5 |
| 203021_at | SLPI |
| 209679_s_at | SMAGP |
| 215714_s_at | SMARCA4 |
| 204240_s_at | SMC2 |
| 34868_at | SMG5 |
| 205309_at | SMPDL3B |
| 218393_s_at | SMU1 |
| 221261_x_at | SNORA11E///SNORA11D///MAGED4///MAGED4B |
| 206055_s_at | SNRPA1 |
| 214708_at | SNTB1 |
| 221561_at | SOAT1 |
| 213456_at | SOSTDC1 |
| 209842_at | SOX10 |
| 204914_s_at | SOX11 |
| 204915_s_at | SOX11 |
| 204913_s_at | SOX11 |
| 213668_s_at | SOX4 |
| 221685_s_at | SPDL1 |
| 213562_s_at | SQLE |
| 204675_at | SRD5A1 |
| 202200_s_at | SRPK1 |
| 200956_s_at | SSRP1 |
| 213355_at | ST3GAL6 |
| 210073_at | ST8SIA1 |
| 202293_at | STAG1 |
| 209969_s_at | STAT1 |
| 217553_at | STEAP1B |
| 212009_s_at | STIP1 |
| 218499_at | STK26 |
| 200783_s_at | STMN1 |
| 209024_s_at | SYNCRIP |
| 210613_s_at | SYNGR1 |
| 212730_at | SYNM |
| 218308_at | TACC3 |
| 203938_s_at | TAF1C |
| 209523_at | TAF2 |
| 210053_at | TAF5 |
| 202307_s_at | TAP1 |
| 206838_at | TBX19 |
| 209153_s_at | TCF3 |
| 209151_x_at | TCF3 |
| 202648_at | TCF3 |
| 221016_s_at | TCF7L1 |
| 212759_s_at | TCF7L2 |
| 205796_at | TCP11L1 |
| 204281_at | TEAD4 |
| 41037_at | TEAD4 |
| 213346_at | TEX30 |
| 205287_s_at | TFAP2C |
| 205286_at | TFAP2C |
| 203589_s_at | TFDP2 |
| 208691_at | TFRC |
| 207332_s_at | TFRC |
| 203092_at | TIMM44 |
| 219258_at | TIPIN |
| 221843_s_at | TLDC1 |
| 203222_s_at | TLE1 |
| 215034_s_at | TM4SF1 |
| 209386_at | TM4SF1 |
| 209387_s_at | TM4SF1 |
| 215033_at | TM4SF1 |
| 211967_at | TMEM123 |
| 213338_at | TMEM158 |
| 209656_s_at | TMEM47 |
| 209655_s_at | TMEM47 |
| 211689_s_at | TMPRSS2 |
| 214051_at | TMSB15B///TMSB15A |
| 207037_at | TNFRSF11A |
| 206641_at | TNFRSF17 |
| 218856_at | TNFRSF21 |
| 214581_x_at | TNFRSF21 |
| 204080_at | TOE1 |
| 201291_s_at | TOP2A |
| 201292_at | TOP2A |
| 203786_s_at | TPD52L1 |
| 213616_at | TPGS2 |
| 212654_at | TPM2 |
| 210052_s_at | TPX2 |
| 205598_at | TRAIP |
| 211002_s_at | TRIM29 |
| 202504_at | TRIM29 |
| 213634_s_at | TRMU |
| 204317_at | TRMU///GTSE1 |
| 206827_s_at | TRPV6 |
| 215442_s_at | TSHR |
| 209109_s_at | TSPAN6 |
| 213122_at | TSPYL5 |
| 204407_at | TTF2 |
| 211714_x_at | TUBB |
| 209191_at | TUBB6 |
| 203690_at | TUBGCP3 |
| 221964_at | TULP3 |
| 202858_at | U2AF1 |
| 205890_s_at | UBD///GABBR1 |
| 202954_at | UBE2C |
| 202779_s_at | UBE2S |
| 201387_s_at | UCHL1 |
| 202330_s_at | UNG |
| 219211_at | USP18 |
| 217829_s_at | USP39 |
| 202549_at | VAPB |
| 201797_s_at | VARS |
| 219740_at | VASH2 |
| 211527_x_at | VEGFA |
| 210512_s_at | VEGFA |
| 215729_s_at | VGLL1 |
| 205487_s_at | VGLL1 |
| 214004_s_at | VGLL4 |
| 209822_s_at | VLDLR |
| 203798_s_at | VSNL1 |
| 200628_s_at | WARS |
| 216228_s_at | WDHD1 |
| 209196_at | WDR46 |
| 218278_at | WDR74 |
| 209053_s_at | WHSC1 |
| 202133_at | WWTR1 |
| 202132_at | WWTR1 |
| 214776_x_at | XYLB |
| 221203_s_at | YEATS2 |
| 219312_s_at | ZBTB10 |
| 214311_at | ZFPL1 |
| 206928_at | ZNF124 |
| 206683_at | ZNF165 |
| 210697_at | ZNF257 |
| 203520_s_at | ZNF318 |
| 220617_s_at | ZNF532 |
| 207120_at | ZNF667 |
| 220967_s_at | ZNF696 |
| 207781_s_at | ZNF711 |
| 219995_s_at | ZNF750 |
| 218349_s_at | ZWILCH |
| 211645_x_at | |
| 216822_x_at | |
| 206548_at | |
| 217036_at | |
| 216484_x_at | |
| AFFX-r2-Bs-dap-M_at | |

Sigma-1 receptor gene: *S1R;* pathologic complete response: pCR; residual disease: RD
