# Supplementary material for: The Sigma‐1 Gene as a Prognostic Marker in Chemotherapy‐Treated Breast Cancer‐Antagonists' Synergism With Paclitaxel In Vitro
Source: Cancer Med. 2025 Nov 12;14(21):e71376. doi: 10.1002/cam4.71376 (PMC12611308; doi:10.1002/cam4.71376)
Supplement: Supplementary file 4 — Table S2: Calculated values for combination indices (CombI) and fraction affected (Fa) as described by the Chou‐Talalay method on MDA‐MB‐231 and HCC1806 cells exposed to Sigma‐1 receptor antagonists IPAG/BD1047 plus Px. [file CAM4-14-e71376-s003.docx]

**Supplementary Table 2:**

Calculated values for combination indices (CombI) and fraction affected (Fa) as described by the Chou-Talalay method on MDA-MB-231 and HCC1806 cells exposed to Sigma-1 receptor antagonists IPAG/BD1047 plus paclitaxel

| **Cell line and combination** | **Treatment concentrations**  **(μM)** | **Fa** | **CombI (Mean ± SEM)** |
| --- | --- | --- | --- |
| **MDA-MB-231**  BD1047 + Px | 20 + 0.1 | 0.52 | 0.96 ± 0.24 |
|  | 50.0 + 0.1 | 0.66 | 0.81 ± 0.1 |
|  | 10.0 + 1.0 | 0.73 | 0.70 ± 0.12 |
|  | 20.0 + 1.0 | 0.75 | 0.71 ± 0.12 |
|  | 100.0 + 0.0001 | 0.79 | 0.87 ± 0.07 |
|  | 100.0 + 0.01 | 0.79 | 0.86 ± 0.11 |
|  | 100.0 + 0.001 | 0.81 | 0.82 ± 0.12 |
|  | 50.0 + 1.0 | 0.83 | 0.56 ± 0.12 |
|  | 100.0 + 0.1 | 0.88 | 0.57 ± 0.07 |
|  | 100.0 + 1.0 | 0.94 | 0.40 ± 0.10 |
| **MDA-MB-231**  IPAG + Px | 1.0 + 0.1 | 0.50 | 2.19 ± 1.71 |
|  | 5.0 + 0.001 | 0.55 | 1.11 ± 0.12 |
|  | 2.0 + 0.1 | 0.61 | 0.55 ± 0.11 |
|  | 5.0 + 0.1 | 0.62 | 1.05 ± 0.16 |
|  | 5.0 + 0.01 | 0.68 | 0.85 ± 0.34 |
|  | 2.0 + 1.0 | 0.76 | 0.6 ± 0.29 |
|  | 1.0 + 1.0 | 0.76 | 0.9 ± 0.33 |
|  | 10.0 + 0.0001 | 0.78 | 1.17 ± 0.24 |
|  | 5.0 + 1.0 | 0.86 | 0.64 ± 0.20 |
|  | 10.0 + 0.1 | 0.89 | 0.6 ± 0.31 |
|  | 10.0 + 0.001 | 0.90 | 0.77 ± 0.26 |
|  | 10.0 + 0.01 | 0.91 | 0.55 ± 0.29 |
|  | 10.0 + 1.0 | 0.93 | 0.5 ± 0.20 |
| **HCC1806**  BD1047 + Px | 50.0 + 0.01 | 0.66 | 1.13 ± 0.35 |
|  | 10.0 + 0.1 | 0.75 | 0.68 ± 0.20 |
|  | 20.0 + 0.1 | 0.77 | 0.71 ± 0.14 |
|  | 50.0 + 0.1 | 0.85 | 0.63 ± 0.07 |
|  | 20.0 + 1.0 | 0.87 | 0.96 ± 0.06 |
|  | 100.0 + 0.0001 | 0.87 | 0.95 ± 0.09 |
|  | 100.0 + 0.001 | 0.88 | 1.47 ± 0.45 |
|  | 10.0 + 1.0 | 0.88 | 1.60 ± 0.95 |
|  | 100.0 + 0.01 | 0.93 | 0.81 ± 0.23 |
|  | 50.0 + 1.0 | 0.93 | 0.64 ± 0.08 |
|  | 100.0 + 0.1 | 0.95 | 0.58 ± 0.10 |
|  | 100.0 + 1.0 | 0.96 | 0.72 ± 0.15 |
| **HCC1806**  IPAG + Px | 1.0 + 0.1 | 0.52 | 2.19 ± 1.71 |
|  | 5.0 + 0.001 | 0.57 | 1.05 ± 0.10 |
|  | 2.0 + 0.1 | 0.63 | 0.55 ± 0.11 |
|  | 5.0 + 0.1 | 0.66 | 1.00 ± 0.12 |
|  | 5.0 + 0.01 | 0.74 | 0.73 ± 0.27 |
|  | 2.0 + 1.0 | 0.75 | 0.90 ± 0.33 |
|  | 1.0 + 1.0 | 0.75 | 0.60 ± 0.18 |
|  | 10.0 + 0.0001 | 0.78 | 1.00 ± 0.12 |
|  | 5.0 + 1.0 | 0.83 | 0.65 ± 0.20 |
|  | 10.0 + 0.001 | 0.85 | 0.72 ± 0.22 |
|  | 10.0 + 0.01 | 0.89 | 0.62 ± 0.36 |
|  | 10.0 + 0.1 | 0.91 | 0.52 ± 0.23 |
|  | 10.0 + 1.0 | 0.94 | 0.37 ± 0.08 |

CombI < 1, = 1, and > 1 indicate synergism, additive effect, and antagonism, respectively. Values were generated through Compusyn software [7].
